# Optimization of biogas yield from lignocellulosic materials with different pretreatment methods: a review

**DOI:** 10.1186/s13068-021-02012-x

**Published:** 2021-07-19

**Authors:** Kehinde Oladoke Olatunji, Noor A. Ahmed, Oyetola Ogunkunle

**Affiliations:** Department of Mechanical Engineering Science, Faculty of Engineering and Built Environment, University of Johannesburg, Johannesburg, South Africa

**Keywords:** Lignocellulose, Hydrolysis, Pretreatments, Biogas, Methane

## Abstract

Population increase and industrialization has resulted in high energy demand and consumptions, and presently, fossil fuels are the major source of staple energy, supplying 80% of the entire consumption. This has contributed immensely to the greenhouse gas emission and leading to global warming, and as a result of this, there is a tremendous urgency to investigate and improve fresh and renewable energy sources worldwide. One of such renewable energy sources is biogas that is generated by anaerobic fermentation that uses different wastes such as agricultural residues, animal manure, and other organic wastes. During anaerobic digestion, hydrolysis of substrates is regarded as the most crucial stage in the process of biogas generation. However, this process is not always efficient because of the domineering stableness of substrates to enzymatic or bacteria assaults, but substrates’ pretreatment before biogas production will enhance biogas production. The principal objective of pretreatments is to ease the accessibility of the enzymes to the lignin, cellulose, and hemicellulose which leads to degradation of the substrates. Hence, the use of pretreatment for catalysis of lignocellulose substrates is beneficial for the production of cost-efficient and eco-friendly process. In this review, we discussed different pretreatment technologies of hydrolysis and their restrictions. The review has shown that different pretreatments have varying effects on lignin, cellulose, and hemicellulose degradation and biogas yield of different substrate and the choice of pretreatment technique will devolve on the intending final products of the process.

## Introduction

Human civilization era was defined with energy utilization; this was the period when prehistoric human understands the use of energy in the form of fire for household satisfaction and cooking. This civilization age then moved to the era of locomotive, the era of nuclear power generation, the automobile, air plane age, the individual computer, and the wireless internet era. All through these centuries, the human life style has developed to the stage where energy utilization is important for the functioning of modern-day society, the success of a nation, and continuous existence of our civilization. The persistent increase in utilization of energy from the beginning of the industrial revolution has led to forceful alteration of the environment globally, the carbon-dioxide concentration present in the atmosphere has increased from 280 ppm in 1750 to above 390 ppm in 2011, and this is one of the major important changes recorded [1]. More than 88% of the principal energy utilized is from fossil fuels [2] and their combustion leads to the release of greenhouse gases, most especially carbon-dioxide [3]. Due to these facts, replacement of fossil fuels with renewable energies gives the opportunity to eradicate these challenges by reducing the persistent rise in temperature globally [4].

**Fig. 1:**
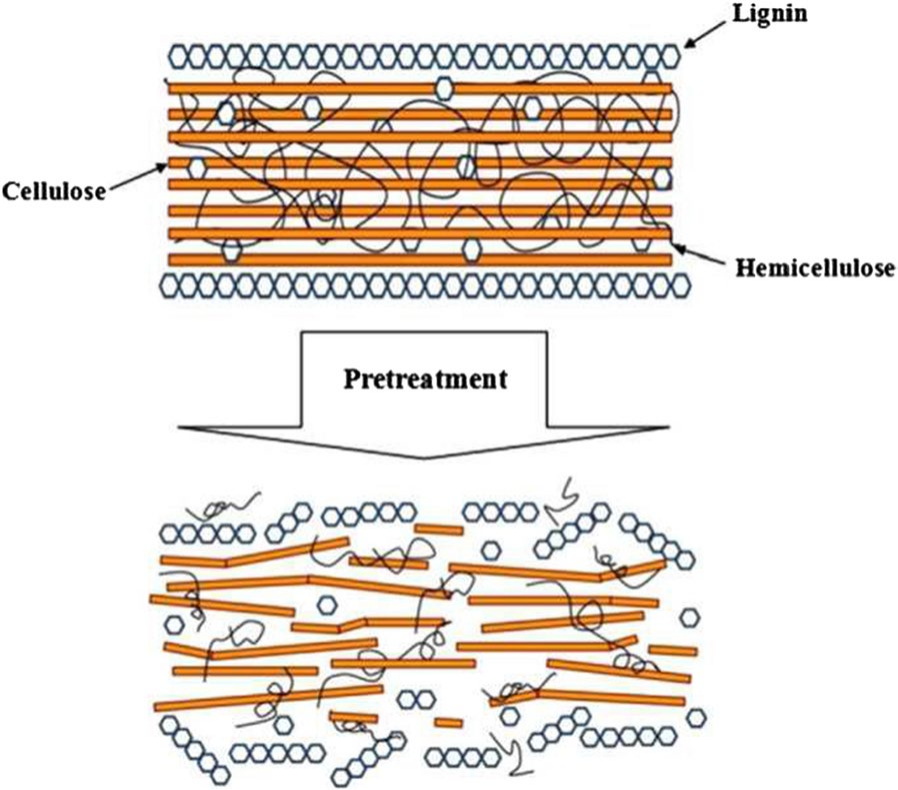
Schematic structure of lignocellulose before and after pretreatment (Source: [12])

Substituting fossil fuels with clean fuel such as biogas for cooking, heating, lighting, and electricity generation will assist in cutting down the greenhouse gas emissions and indoor air pollution [5]. The uses of biomass as renewable energy sources through different technologies is regarded as sustainable technology to meet up with the energy required and also reduce the release of greenhouse gases. Furthermore, biomass utilization gives the benefit of cost-efficient viability and minimizes the quantity of waste released to the environment [6]. Biogas, the product of anaerobic digestion of biomass can be employed as a changeable energy sources for electricity and heat generation, either separately or blended, and to drive vehicles. Biogas generation have some advantages such as organic waste control, cutting down of greenhouse gas, and production of viable fertilizer at a reasonable cost [7, 8]. During anaerobic digestion, complicated polymers are digested with homogenous molecules and into biogas that is mostly 60–70% CH_4_ and 30–40% CO_2_ [9] and some other gases in traces. The quantity of CO_2_ during the composition of biomass is equivalent to the same amount of carbon needed by the plant during photosynthesis and this is an advantage that classifies biomass as carbon neutral [10]. Biomasses are readily available globally as agricultural residues/wastes and residual wastes. The most crucial and abounding renewable feedstock sources include crop residues like rice straw, maize cob, corn straw, groundnut shell, wheat straw, etc. and animal wastes. Lignocellulosic substrates from crops residues are the principal feedstock for biogas generation and this process assists in effective waste management and as the major renewable bioenergy sources. In spite of the potential of this feedstock for biogas production, they have a complex compositional and structural arrangement that have high resistance to biological degradation, a characteristic that is referred to as biomass recalcitrance. There are three main biopolymers that are present in lignocellulosic materials, and they are cellulose, hemicellulose, and lignin (Fig. 1). The microfibrils of cellulose are confined in a matrix of intertwined hemicellulose and lignin referred to as lignin–carbohydrate complex, forming a resistance to effective biological decomposition [11]. Therefore, there is need for techniques to lower the biomass recalcitrance and thus enhance the availability of lignocellulosic materials to anaerobic microbial decomposition.

Pretreatment is a crucial technology for cellulose transformation process, and it is important to alter the arrangement of cellulosic feedstock to allow cellulose to be more useable by the enzymes that produce fermentable sugars from carbohydrate polymers [13]. Pretreatment modifies the different feedstock structures at all fiber levels. The degree and proportion of lignocellulosic materials hydrolysis and morphological characteristics are altered by biological, physical, chemical, and thermal pretreatment. Notwithstanding, the most economical and effective techniques among all these technologies have not been established yet. Also, the optimum considerations for pretreatment are not always mentioned. These observations are important for the effective and practicable use of various residues available from agricultural activities [14]. The focus of this paper is to review pretreatment methods for biogas generation from agricultural residues and to present an in-depth discourse on the advantages and drawback of the methods in biogas production.

## Nature of agricultural residues/wastes

Agricultural residues are majorly plant and animal residues. Plant cells are completely enclosed in cell membrane and one or two cell walls which depend on the plants type. The outside wall that protects the cell is primary cell wall, while the secondary cell wall is in between the primary cell wall and the cell membrane. Polysaccharide which is mainly pectin lies between the walls and bind the cells together. Primary cell wall is more flexible because of its different composition when compared with secondary cell wall. The plant polysaccharides can be divided into cellulose and hemicellulose and they are sugar polymers that can be fermented into sugars needed in biogas production, while lignin can be converted into chemicals. In general, crop residues like wheat straw, corn cob, sugarcane bagasse, sorghum stalk, rice straw, groundnut shell, corn stover, etc., contain substantial amount of lignocellulosic contents and have been proved to be a viable feedstock for biogas generation, but wastes from fruits and vegetables and grasses have lesser lignocellulosic content. Table 1 shows the composition of some lignocellulosic materials, and on a dry basis, biomass is usually cellulose 50%, hemicellulose (10–30% in woods or 20–40% in herbaceous biomass), and lignin (20–40% in woods and 10–40% in herbaceous biomass) [15]. Nevertheless, this content of cellulose, hemicellulose, and lignin ratio may vary because of variation in age, conditions of the culture, and harvesting season. Lignin in particular resists the enzymatic debasement of materials with high-crystalline structure and made them insoluble in water, because lignin and hemicellulose produce a protective sheath around the cellulose. Lignin plays an important role in inhibiting the degradation of the hemicellulose and cellulose feedstock to monomeric sugars that is required for efficient transformation of feedstock to biofuels. Hence, for efficient production of energies like biogas, methanol, bio-ethanol, etc. from lignocellulose materials, it essential to pretreat the feed stock.

**Table 1:** Percentage cellulose, hemicellulose, and lignin content of some lignocellulosic feedstocks. Adapted from [16]

S/N	Materials	Cellulose (%)	Hemicellulose (%)	Lignin (%)
1	Pine	45.60	24.00	26.80
2	Groundnut shell	37.00	18.70	28.00
3	Rubber wood	39.56	28.42	27.58
4	Hardwood Eucalyptus	44.90	28.90	26.20
5	Softwood Spruce	47.10	22.30	29.20
6	Grasses Bamboo	46.50	18.80	25.70
7	Reed	49.40	31.50	8.74
8	Oak	43.20	21.90	35.40
9	Rye	42.83	27.86	6.51
10	Walnut shell	23.30	20.40	53.50
11	Sunflower	34.06	5.18	7.72
12	Japanese cedar	52.70	13.80	33.50
13	Silage	39.27	25.96	9.02
14	Szarvasi-1	37.85	27.33	9.65
15	Hemp	53.86	10.60	8.76
16	Pine nut shell	31.00	25.00	38.00
17	Cotton stalk	67.00	16.00	13.00
18	Natural hay	44.90	31.40	12.00
19	Hemp stalk	52.00	25.00	17.00
20	Amur silver-grass	42.00	30.15	7.00
21	Coconut coir	44.20	22.10	32.80
22	Acacia pruning	49.00	13.00	32.00
23	Rice husk	40.00	16.00	26.00
24	Rice straw	38.14	31.12	26.35
25	Bamboo leaves	34.14	25.55	35.03
26	Extracted olive pomace	19.00	22.00	40.00
27	Palm oil frond	37.32	31.89	26.05
28	Sugarcane peel	41.11	26.40	24.31
29	Hazel branches	30.80	15.90	19.90
30	Barley straw	35.40	28.70	13.10
31	Corn stover	43.97	28.94	21.82
32	Pistachio shell	15.20	38.20	29.40
33	Coffee grounds	33.10	30.03	24.52
34	Almond shell	27.00	30.00	36.00
35	Hazelnut shell	30.00	23.00	38.00

Lignocellulosic materials’ pretreatment is an influential step in the transformation of biomass into fermentable sugars, and it paves way for hydrolysis stage where lignin and hemicellulose components are break down to release the cellulose buried in it [17]. Pretreatment techniques must be simple, eco-friendly, feasible, and economical [18]. In the same vein, pretreatment techniques must not result in the production of inhibitory compounds or loss of lignin and polysaccharide. Moreover, up to date, there is no reconciled pretreatment technique that is suitable for all type of lignocellulose materials and the expected outputs. Nevertheless, combining two or more techniques can improve the effectiveness of the process significantly and bring about the breakthrough in this area of study.

## Biogas production

**Fig. 2:**
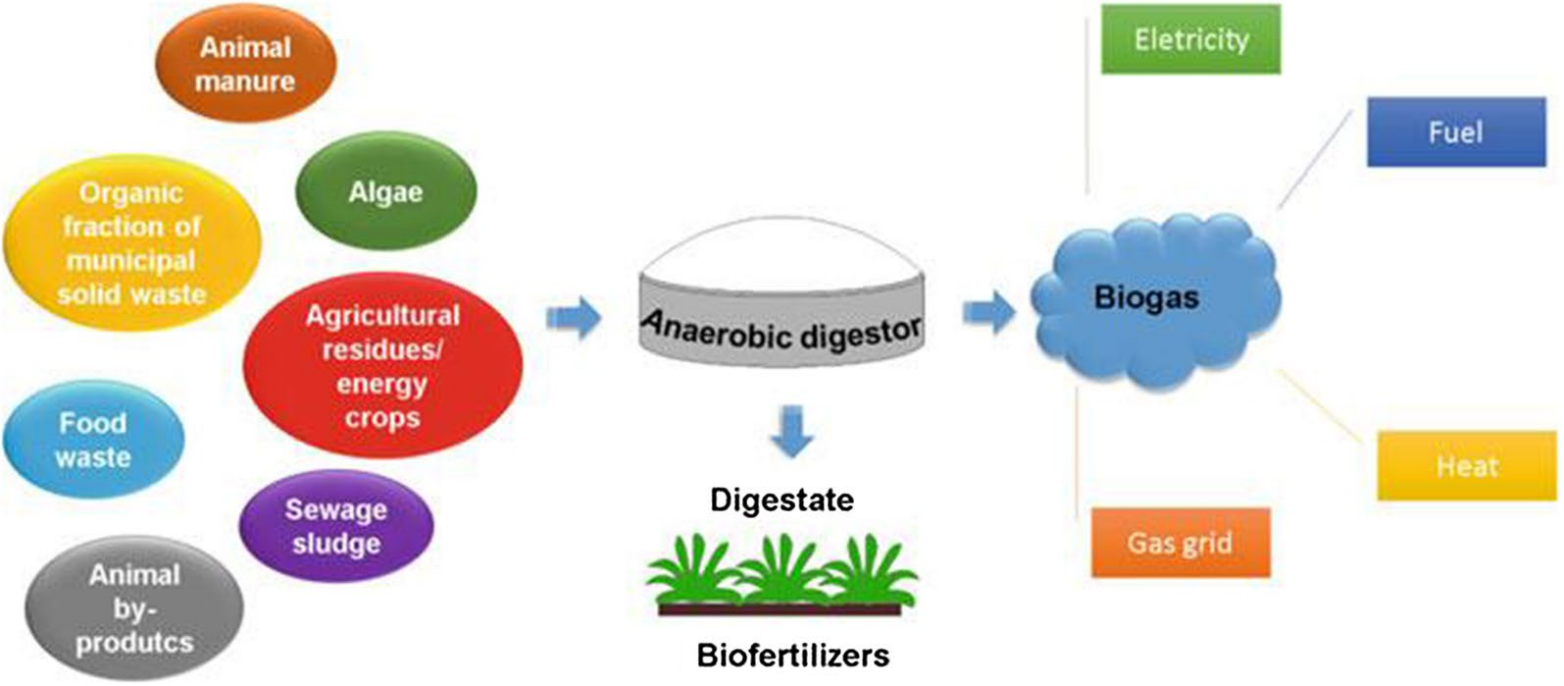
Conventional diagram of biogas generation and utilization (Source: [24])

Biogas is a biofuels’ type that originates from biodegradable materials typically a gas released by catalytic action of fermentation bacterial on organic materials in the absence of oxygen (anaerobic digestion condition). It is the result of well-arranged biologically intervened system resulting from microorganisms digestion of plant and/or animal in airtight containers called digester. Biogas is a composition of mainly methane (CH_4_) about 50–70% and carbo-dioxide (CO_2_) of about 30–50%, and the percentage of methane and carbon-dioxide present in the biogas mixture is determined majorly by the type of the feedstock [19]. Aside from methane and carbon-dioxide, there is traces of N_2_ (0–3%), H_2_S (0–10,000 ppm), O_2_ (0–1%), H_2_O (5–10%), and NH_3_ in the mixture [19, 20]. Biogas production is an alluring alternative energy source with regards to energy yield. The cumulative biomass supply in 2014 was calculated to be 59.2 EJ which represents about 10.3% of the total energy supplied globally [21]. Agriculture, forestry, and organic fraction of the municipal solid waste contributed 10, 87, and 3%, respectively to the biomass supplied [22]. Biogas produced can be employed to produce electricity, heat, and for engines use (Fig. 2), fuel cells, and micro-turbine. It can also be upgraded into biomethane which is referred to as Renewable Natural Gas (RNG) and utilized in transport sector or injected into the gas grid [23].

**Fig. 3:**
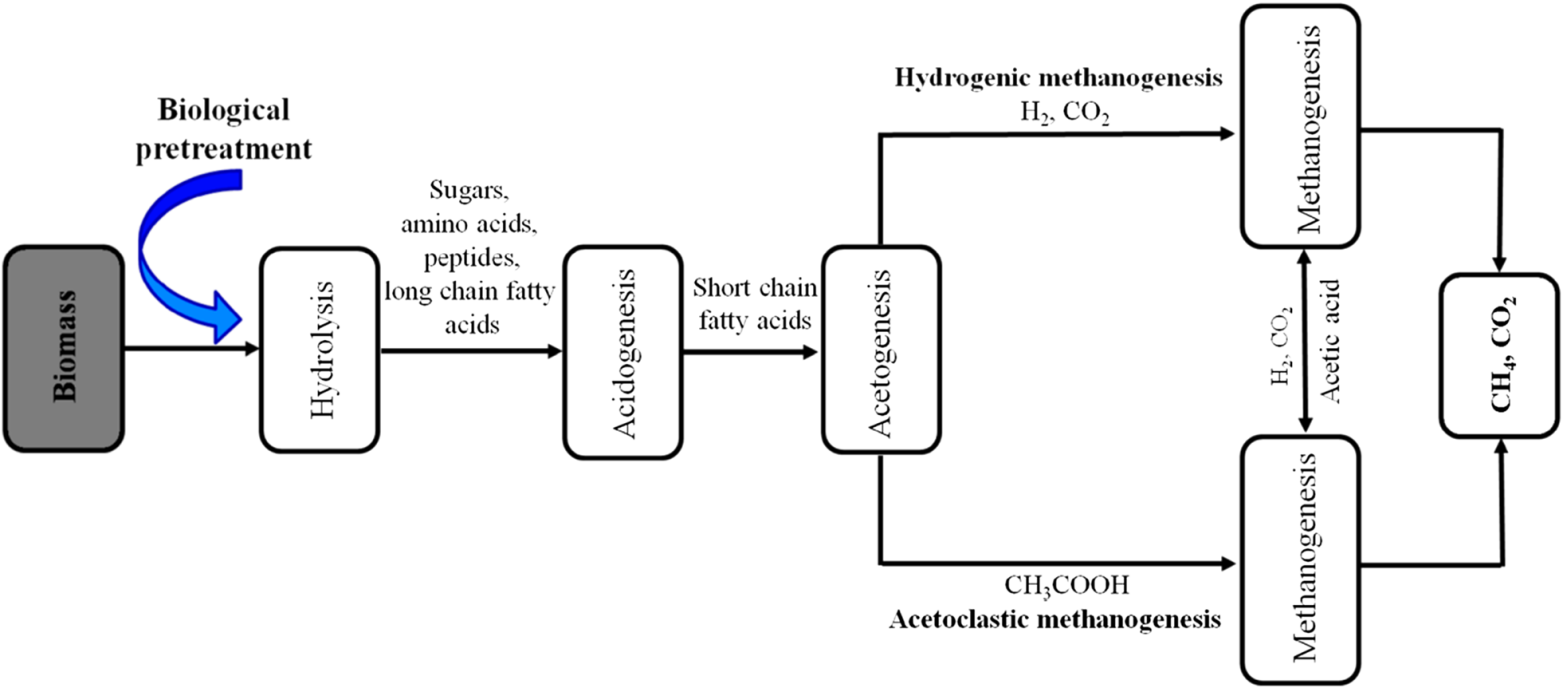
A flowchart showing the biological stages of anaerobic digestion

Various types of anaerobic digesters have been designed, constructed, and used for biogas production, but irrespective of the digester type employed, there is need to monitor the performance of the configuration in use thoroughly to forbid any sudden change that may occur during the process. Production parameters like temperature, hydraulic retention time, pH, total solid, volatile fatty acids, volatile solid, organic loading rate, shear stress, mixing, and inhibitors (NH_3_, hydroxymethyl furfural, furfural, etc.) can alter the process adversely and affect the effectiveness of the process if not well managed. Therefore, a proper range of these parameters must be set during the biogas production process to have a favorable biogas production process and yield [25]. Mesophilic (35–40 °C) and thermophilic (55–60 °C) are the two optimum temperature ranges for anaerobic digestion. Majority of the anaerobic digestion plant in the globe operate at mesophilic range, because heat required to stabilize that temperature is low and the process is relatively stable in this temperature range. On the other hand, thermophilic plants required higher heat and needed more attention to operate, but they are needed when digestion needs to be accelerated and resulting into improved biogas yields and lower the pathogens in effluent slurry [26]. Hydrolysis, acidogenesis, acetogenesis, and methanogenesis are the four biological and chemical steps of anaerobic digestion (Fig. 3).

Hydrolysis is the stage where large organic polymers of biomass such as carbohydrates, proteins, and fats are reduced to smaller molecules like simple sugars, amino acids, and fatty acids. It is a rate constrictive step as it determines the rate of biodigestion of the feedstock an important step of anaerobic digestion where complex organic matters are hydrolyzed into soluble molecules by the catalytic action of the fermentative bacteria. Hydrogen and acetate that will be utilized by methanogens at the later stage of the process are the products of hydrolysis, while some of the molecules that may still be relatively large have to be broken further during acidogenesis to be useful during methane production [27]. The second stage of anaerobic digestion is acidogenesis; at this stage, acidogenic bacterial break down the feedstock from hydrolysis further. These fermentative microorganisms generate an acidic environment in the reactor and produce NH_3_, CO_2_, H_2_, H_2_S, organic acids, and shorter volatile acids with some quantity of other by-products in traces. Some of the major acids released in this stage are butyric acid, acetic acid, propionic acid, etc. Acetogenesis is the anaerobic digestion stage where acetate a derivative of acetic acid is produced from carbon and energy sources by bacterial called acetogens. Acetogens catabolize some of the products of acidogenesis stage into acetic acid, CO_2_, and H_2_ and also break down the feedstock to the level; it will be useful for methanogens during methane production. The last and the final stage of anaerobic digestion is methanogenesis; the end-products of acetogenesis are transformed into methane by the methanogens. This stage has two general pathways (Eqs. 1 and 2) that involve the utilization of the acetic acid and carbon-dioxide that are the two major products of the first three stages of anaerobic digestion to release methane during methanogenesis stage. CO_2_ can be transformed into methane and water (Eq. 1) through the process, while the major mechanism to liberate methane during methanogenesis is the path involving acetic acid (Eq. 2) and this leads to the liberation of methane and carbon-dioxide, the two major products of anaerobic digestion [27]

1 $${\text{CO}_{2}} + 4{\text{H}_{2}} \to {\text{CH}_{4}} + 2{\text{H}_{2}} \text{O}$$

2 $${\text{CH}_{3}} \text{COOH} \to {\text{CH}_{4}} + {\text{CO}_{2}}.$$

## Optimization of biogas and methane yields

Biogas yield can be improved with different means as reported by different authors. This includes: pretreatments [28–31], co-digestion [32–35], bioaugmentation [36–38], biohythane [39–41], temperature, organic loading rate, and reactor design [42–45].

### Pretreatment of substrate for methane production

Different factors such as lignin percentage, crystallinity, polymerization grade, surface area, and solubility determine the degradability of lignocellulose feedstock [40]. Different researchers have examined the application of different pretreatment techniques to enhance the biodigestion of lignocellulosic feedstock and enhance methane release. The selection of pretreatment technique depends on the physicochemical characteristics and structural arrangement of the feedstock; and it is expected to improve the formation of organic feedstock and still maintain the matter in the process. Biological, chemical, and physical pretreatment techniques include enzyme, fungi, acid, alkali, ionic liquids, organosolvents, ozonolysis, size reduction, extrusion, steam explosion, liquid hot water, etc. [46]. Pretreatment of substrate before anaerobic digestion has the same objectives with pretreatment before ethanol production, the only difference is that since the microorganisms is involved, anaerobic digestion is able to breakdown crystalline cellulose structures and hemicelluloses; and pretreatment can be less expensive.

The breaking down of the lignin-polysaccharide bonds and the opening of the materials are the two main focus of pretreatment prior to anaerobic digestion [11]. In general, the objectives of pretreatments are to

i. ease the approachability of the enzymes to the cellulose and hemicelluloses and lead to degradation of the feedstock;
ii. avoid degradation or carbohydrates loss;
iii. eliminate the release of possible inhibitors;
iv. be economical, and
v. reduce the possible impact on the environment [47].

#### Physical/mechanical pretreatment

Physical/mechanical pretreatment of lignocellulose feedstock is an essential step in enhancing the biodigestion ability, particle compaction and arrangement, enzymatic accessibility, and total conversion of lignocellulosic feedstock into biogas without the production of toxic substance [48]. This technique also produces new surface area, enhances flow characteristics; and improves the porosity and bulk density of the materials.

##### Milling or size reduction

Cellulose crystallinity can be reduced with the use of mechanical milling/grinding which comprise of milling, grinding, and chipping methods. Substrate sizes of 10–30 mm only can be achieved in chipping, while lower particle sizes of up to 0.2 mm can be achieved with grinding and milling [49]. The main focus of the size reduction is to reduce feedstock particle size [11]. This improves the surface area of the substrate and reduces the level of polymerization [50]. Some pretreatment methods required size reduction of the feed stock to a particular level before pretreatment [51]. Milling or size reduction can be the only pretreatment method for some lignocellulosic-rich materials that are easy to degrade. The milling type and duration, and feedstock structure will influence the improvement in particular surface area, net polymerization level, and final cellulose crystallinity reduction. There are different milling techniques (hammer, vibratory, colloid, and two-roll milling) and all of them can be employed to increase the biodigestion of lignocellulosic feedstock [50]. Vibratory ball milling has been adjudged to be the most efficient in cellulose crystallinity reduction and increase the biodigestion of spruce and aspen chips when compared with ordinary milling process. Likewise, wet disk milling has been a preferred mechanical pretreatment technique, because energy required is low. Disk milling increases cellulose hydrolysis by generating fibers and is more efficient in comparison with hammer milling that generates finer particles [52]. Jekayinfa et al*.* [28] reported that different size of groundnut shell had different effects on biogas yields and there is a specific size bound where size reduction of groundnut shells will have negative effects on the biogas release. Rice straw treated with size reduction showed an increase in methane production; however, combining other pretreatment methods with size reduction will give better results [53]. About 5–25% improvement in methane released was recorded when municipal solid wastes was pretreated with size reduction [29]. Spruce milled released six times higher methane in comparison to spruce chip, whereas spruce milled pretreated with *N*-methyimorpholine-*N*-oxide (NNMO) gives 200% higher methane content when compared with spruce chips [54]. The adverse effect of extensive milling technique is high energy required which leads to higher pretreatment costs and makes it inappropriate in some cases [55]. Nevertheless, studies have shown that size reduction less than 0.4 mm had no noticeable influence on hydrolysis rate and biogas released [49].

##### Extrusion

In extrusion pretreatment, feedstock is allowed to undergo heat, compression, and shear force, and this leads to physical destruction and chemical modification of the feedstock while going through the extruder. Extruder design has single or twin screw that twists into a firm barrel that has temperature control apparatus. The feedstock experience friction and energetic shearing that leads to increase in pressure and temperature when pass through the barrel. At the exit of the barrel, the feedstock will experience pressure release and this will result in structural alteration of the feedstock which will enhance biodigestion during the subsequent process [56]. Extrusion of pelleted hay for optimization of biogas released was investigated by Maroušek [57], and it was reported that optimum biogas yield of 405 m^3^/ton TS that has 52.3% methane and 33% improvement in biogas yield when compared with control was recorded when pressure was 1.3 MPa with reaction time of 7 min and 8% dry matter. In a related research, organic fraction of the municipal solid waste was pretreated with extrusion method, improved biogas released of 800 L/kg VS which was about 60% methane content was reported by Novarino and Zanetti [58].

##### Ultrasound

Ultrasonic treatment is a technology that break down and destructs the feedstock, for example, treatment of waste-water sludge particles. Sludge properties, frequency, the energy level, and the time determine the efficiency of ultrasound pretreatment. The aim of this method is to burst microbial cell arrangement and extract cellular material from the cells. In this technique, the morphology of the lignocellulosic feedstock was altered with the physical and chemical effects from ultrasound waves. Ultrasound pretreatment produces little cavitation bubbles that burst the cellulose and hemicellulose portion and improve the availability of degrading enzymes to the cellulose for efficient dislocation into simpler homogenous sugars. The optimum cavitation was produced at 50 °C which is also the highest temperature for some cellulose hydrolytic enzymes [59]. The ultrasonic frequency and time majorly influenced the ultrasonic field; digester size, type, and solvent utilized, and sonication time has major influence on feedstock pretreatment. Nevertheless, sonication above a particular frequency and reaction time has no further influence in terms of debasement and sugar liberation [60]. Pretreatment with ultrasound at frequency of 10–100 kHz has been studied by some researchers and was reported to be sufficient for lignocellulose degradation and polymer debasement [61]. Pretreatment process was reported to have been affected adversely when sonication with higher power level is employed, because this led to bubbles formation close to the top of ultrasound transducer and impedes the transmission of energy to the liquid medium [61]. Three different sonication times of 9, 18, and 27 min with 80 μm amplitude and 20 kHz frequency were examined on fruits and vegetables waste, and the optimum methane yield was recorded at 18 min sonication with specific energy of 2380 kJ/kg total solids (TS) for 20 day period in batch digester, while exposure to sonication for longer period result into lesser methane yield. The energy content of the biogas recorded as at that time was the double of the energy used for sonication [62]. Macro algae was pretreated with sonication at a particular input energy of 75 MJ/kg TS and only 20% of the methane was released, but when the sonication energy was increased to 100–200 MJ/kg TS, methane yield was increased to between 80 and 90% [63]. About 50% of biogas yield increase was recorded when ultrasound was used at full-scale sewage sludge plant [64]. When ultrasound was combined with alkaline pretreatment for thickened pulp mill waste, there was a notable improvement in the initial digestion, but the total methane yield was not improved [65]. This implies that all the input factors in this method are important during optimization of biogas with ultrasound pretreatment.

##### High hydrostatic pressure (HHP)

High hydrostatic pressure works with two basic principles which are: (1) pressure is shared relatively in all areas of the feedstock regardless of the form and size; and (2) the pressure favors all the entire structural reactions and alterations that include reduction in volume. Changes in pressure alteration are not always put into consideration by the researchers, unlike temperature that is a thermodynamic parameter of enzyme-catalyzed reaction. This method does not depend on time/mass and this makes it an advantage when compared with thermal treatment. In addition, the pressure affects only the hydrogen bonds, while the covalent bonds remain unaltered, thereby processing time is reduced. The applied pressure alters the structure of the enzymes and influences their activities, and this changes their reaction mechanism and alteration to the physical arrangements of the feedstock [66]. It has been recorded that high hydrostatic pressure is a bright technique for the pretreatment of lignocellulosic materials to achieve yields with a specified properties, because exposure time and pressure can be used to regulate the proportion and level of enzymatic hydrolysis. Eucalyptus globulus kraft pulp was studied under high hydrostatic pressure of 300–400 MPa for the duration of 15–45 min and it was reported that 5–10-fold improvement in the original hydrolysis rate of xylan by xylanase was noticed after pretreatment [67]. High hydrostatic pressure of up to 400–800 MPa was used to pretreat sugarcane bagasse with combination of various concentrations of chemical compounds. Significant improvement in the susceptibility of feedstock to enzymatic hydrolysis and increase in the concentrations of glucose were reported [68]. The results displayed some cracks, small holes, and some fragments that were flaked off from the compressed lignocellulosic structure with the high hydrostatic pressure treatments at the higher effective pressure of 250 MPa. Albuquerque et al. [69] also reported that hydrolytic characteristics of fungal cellulases on coconut husk feedstock were improved by a factor of 2 when treated under high hydrostatic pressure. The findings showed rupturing and porous areas on the coconut fibers that was pretreated with 300 MPa pressure for the duration of 30 min. High hydrostatic pressure is a bright option not only for biomass pretreatment but also for inducing hydrolytic enzymes stability and activation [70].

##### Gamma ray irradiation

Gamma ray is generated from radioisotopes (Cesium-137 or Cobalt-60) and has been experimented as pretreatment for lignocellulosic materials. Ionizing radiation can pierce into the lignocellulosic materials easily and alter the lignin arrangement; and dislocate the cellulose crystal areas. The aftermath impact is facilitated by the release of free radicals that decay faster from the amorphous areas when the radiation is terminated, while decay at a particular period from the crystalline areas also leads to further debasement of the lignocellulose materials [71]. The impact of γ-irradiation on the biotransformation effectiveness of microcrystalline cellulose (MCC) was investigated by Li et al. [72], and compared with other methods of pretreatment like 1% HCl, ionic liquids, H_2_SO_4_, and acidic aqueous ionic liquids. It was recorded that the most efficient irradiation dose (891 kGy) had about the same efficiency of MCC biotransformation with ionic liquid pretreatment and above other pretreatment methods considered. Several researchers have reported that γ-irradiation pretreatment can improve the enzymatic hydrolysis of lignocellulosic materials [73–76] and the method is a promising pretreatment technology. Rapeseed straw was pretreated with gamma irradiation at 1200 kGy and it was reported that there were series of alteration in physical and chemical characteristics of the biomass. This includes the change in the linkage between the carbohydrates and links within the biomass, specific surface area increase, reduction of the distribution range, particle-size decrease, and reduction in thermal durability of the biomass considered [77]. Improvement of 22% in biogas yield was recorded from sewage sludge cell lysis with γ-irradiation pretreatment [78].

##### Electron beam (EB) irradiation

Electron beam ionizing radiation is generated from a linear accelerator. Accelerated beams’ electrons can be used to irradiate lignocellulose biomass as a means to disrupt the arrangement of the lignin, cellulose, and hemicellulose, and produce radicals that can move freely, disrupt cross-link arrangement or chain scission, decrystallization, and/or lower the extent of polymerization [79]. Dosages of up to 1000 kGy of electron beam irradiation were used to pretreat sugar maple and it was observed that cellulose and hemicellulose arrangements were depolymerize at different degrees, and phenolic yield was improved [80]. Electron beam irradiation of 500 kGy was reported to be the optimum when Korean *Miscanthus sinensis* was pretreated before enzymatic hydrolysis for fermentable sugar production [81]. The method is mostly efficient on depolymerizing of cellulose and this necessitated its use in combination with other techniques like alkali or steam explosion for lignin and hemicellulose hydrolysis [82, 83].

##### Microwave irradiation

Microwave is an electromagnetic radiation that has wavelengths between 1 mm and 1 m, and they are found in between 300 and 300,000 MHz on the electromagnetic spectrum and they are non-ionizing radiation that conveys energy selectively to separate substances [84]. This method is used to break down the cell wall and the cellulosic crystallinity, and improves the available surface area [47]. It has drawn renewed concern in the last 3 decades when hydrolysis, esterification, oxidation, and alkylation processes were improved with the use of microwave heating [85]. Furthermore, adding mild-alkali reagents to the process improved the effectiveness of the process. Several researchers have investigated the use of microwave radiation pretreatment of lignocellulose materials during this period and the results were positive, and this method has moved step by step to pilot scale from laboratory scale [86]. Presently, microwave irradiation pretreatment of lignocellulosic materials can be categorized into two major groups which are microwave-assisted solvolysis and microwave-assisted pyrolysis. Microwave-assisted solvolysis is carried out under bland temperatures (< 200 °C) that depolymerizes the materials to release value-added chemicals, while microwave-assisted pyrolysis is the pretreatment of lignin in the absence of oxygen with higher temperatures (above 400 °C) to transform biomass into bio-oil or bio-gases. These two classes of microwave techniques can be achieved with the use of catalysts. Although, microwave-assisted pyrolysis is the most common type because of the energy shortage and sustainability plans of many countries. Microwave radiation has many benefits like fast heat transfer and lesser reaction period, uniform and selective volumetric heating performance, low degradation or formation of side products, ease of operation, and effectiveness when compared with conventional heating. Higher percentage of acetyl groups present in hemicellulose was removed with the use of microwave hydrothermal treatment and this can be raised from the hot spot effect of microwave irradiation [87]. Dielectric characteristics of some wastes from agriculture and agro-industrial based industries (rice husk, oil palm shell, coconut shell empty fruit bunch, and sawdust) were investigated by Salema et al. [88] and it was discovered that they all have low loss dielectric materials.

Microwave heating has been acknowledged to improve enzymatic saccharification through the swelling of the fiber and fragmentation due to its uniformity in internal and rapid heating of huge feedstock particles [89]. When plant fiber feedstock was pretreated with microwaves at temperatures of up to 100 °C, there was no significant effect on the feedstock [90]. Chen et al. [91, 92] reported higher lignocellulosic structure breakdown when bagasse was pretreated with microwave heating at 190 °C for 5 min. Microwave pretreatment effectiveness depends mostly on the dielectric characteristics of the feedstock which depict the strength of the materials to stock electromagnetic energy and transform it to heat. Although, biomass is normally absorbing by microwave at a very low rate and this can be improved with comparatively high moisture and inorganic content of the biomass [93]. Increasing the commercial accessibility of flow-through microwave pretreatment can be of specific importance to the pretreatment of lignocellulose materials. Microwave pretreatment of sorghum bagasse was experimented by Choudhary et al. [94] and it was reported that around 65% of the maximum total sugars were reclaimed when 1 g of sorghum bagasse put in 10 mL of water was exposed to 1000 W for the duration of 4 min. Rape straw was treated with microwave at various energy and time; higher glucose was recorded at higher energy, but at a particular energy setting, treatment duration had no significant impact [95]. Rice husk and corn straw in alkaline glycerol was pretreated with microwave-assisted pretreatment and examined under the scanning electron microscope, and the result showed significant interruption of the plant cell arrangement [89]. Spent grain from the breweries was pretreated with microwave-assisted alkali (1 g of the spent grain in 10 mL of 0.5% NaOH using 400 W for the period of 60 s) and compared with steam explosion, dilute acid hydrolysis, ferric chloride, ammonia fiber explosion, and organosol pretreatment, and it was recorded that the microwave-assisted alkali pretreatment was the most suitable method [31]. The spent grain treated with microwave-assisted alkali produced 228.25 mg of reducing sugar/g of spent grains which was better than the untreated spent grain by 2.86-folds (79.67 mg/g of spent grain). Switch grass pretreatment with microwave-based alkali pretreatment was reported to have produced about 70–90% sugars [36] and sodium hydroxide was reported to be the best alkali. When switch grass and coastal Bermuda grass were pretreated with microwave-based alkali technique with different alkali, at optimum conditions, 82% glucose and 63% xylose was recorded from switch grass, while 87% glucose and 59% xylose were recorded for coastal Bermuda grass [96]. Though the difference is not significant, the researchers has related the difference in reducing sugars to the variation in the percentage of lignin (switch grass 22% and Bermuda 19%) present in these lignocellulosic feedstock. In another related research, Zhu et al. [97–99] examined chemically pretreated *Miscanthus* with microwave technique. Microwave was used to pretreat the *Miscanthus* already pretreated with NaOH and H_2_SO_4_ and it was reported that 12 times higher sugar released was recorded in half the time when compared with the usual heating with H_2_SO_4_ and NaOH techniques. This was mainly as a consequence of the pre-interruption of crystalline cellulose and lignin solubilization with chemical pretreatment. Microwave irradiation pretreatment of lignocellulose materials has been reported to have the benefit of easy of operation, not capital intensive, and visible energy effectiveness [100].

##### Pulsed‑electric field

Pretreatment with pulse electric field (PEF) is aimed at exposing the cellulose in the feedstock by open the holes within the cell membrane and allow accessibility of the agents that will split the cellulose into constituent sugars. During PEF pretreatment, the feedstock is exposed to an abrupt burst of high voltage of between 5.0 and 20.0 kV/cm for a short period of time (nanoseconds to milliseconds). The benefit of PEP is the little energy required as a result of short period (100 µs) of pulse duration, and it can be performed at ambient conditions. Likewise, the PEF equipment has simple design, because it does not have moving parts [101]. Luengo et al. [102] investigated two separate time range of milliseconds and microseconds to pretreat *Chlorella vulgaris*, and at > 4 kV/cm electroporation was found to be irreversible in the millisecond range and at ≥ 10 kV/cm in the microseconds range. Pig slurry and waste activated sludge were pretreated with pulse electric field and methane produced was increased by 80% for pig slurry and methane yield from the sludge was improved by twofold [103]. For this pretreatment method, there is need for more studies to establish further the effectiveness of pulsed electric fields on the structure of lignocellulose materials.

##### High-pressure homogenization (HPH)

High-pressure homogenization is a very popular mechanical pretreatment techniques used for cell interruption and retrieval of intracellular bio-products. The homogenizer is equipped regarding the production of homogenous size arrangement of particles delayed in the liquid, by utilizing a pressure pump to force the liquid through a particular valve to accomplish homogenization. The operating pressure determines the type of the system; when the pressure is about 150–200 MPa, it is referred to as high-pressure homogenization (HPH), while at pressure of up to 350–400 MPa is known as ultra-high-pressure homogenization (UHPH) [104]. Lignocellulosic materials of grass clipping, catalpa, corn straw, and pine sawdust were pretreated with HPH under 10 MPa working pressure. It was observed that there was reduction in particle size of the biomass and the accessible area for enzymatic hydrolysis was improved. This results into increase in the yield of reducing sugars [105]. In comparison with alkaline-heat pretreatment, HPH pretreatment is a bright environmental benign technique for biogas generation from lignocellulose materials. It can rearrange the micro-arrangement of lignocellulose materials to an “empty-inside” arrangement and improve the enzymatic attack without losing the hemicellulose content [105]. Sugarcane bagasse was pretreated with HPH at 100 MPa, the particle size was decrease significantly, and interruption in the micro-arrangement of the biomass improved the available surface area by threefolds [106]. Nano-fibrillated cellulose has been separated from lignocellulose biomass with the use of this highly effective, simple, and green mechanical homogenization [106].

Biogas yield from different lignocellulose materials that were pretreated mechanically is as shown in Table 2. The table shows that there is no particular particle size of lignocellulose materials that is applicable for the optimum biogas yield of all the available feedstock. It can be observed that when the particle size of water hyacinth was reduced from 1.0 to 0.05 mm, the yield increased from 10 to 16% [107], whereas when wheat straw particle size was reduced from 1.2 to 0.3 mm, there was a decrease of 19.1 mL/g VS in the biogas yield [108]. It can also be inferred that at 0.3 mm particle size, different biomass produced different result. There was an increase of 4.6 mL/g Vs when rice straw was pretreated to 0.3 mm particle size [109], while the biogas yield was increased by 77.8 mL/g VS when wheat straw was pretreated to the same 0.3 mm particle size [108]. Likewise, it was shown that different mechanical pretreatment methods also have different effects on the same lignocellulose biomass. Wheat straw was treated with two different mechanical methods of size reduction and high hydrostatic pressure, and the results indicated that the biogas yield was improved by 22.40% for size reduction [108], while the biogas yield from high hydrostatic pressure treatment was increased by 42.02% [110]. Although, this can still be research further with the use of the same bio-digester.

**Table 2:** Different mechanical pretreatment applied to the biogas production and yield

S/N	Biomass	Pretreatment	Anaerobic digestion condition	*Y*_BP_	*Y*_AP_	Refs
1	Water hyacinth	0.05 mm	Digester 0.45 L		Increase by 16%	[107]
2	Water hyacinth	1.0 mm	Digester 0.45 L		Increase by 10%	[107]
3	Meadow grass	200 mm	Bottle 0.5 L	297 mL/g VS	376 mL/g VS	[111]
4	Rice straw	0.3 mm	Glass reactor 2 L	58.1 mL/g VS	62.7 mL/g VS	[109]
5	Wheat straw	0.3 mm	Reactor 2 L	167.8 mL/g VS	245.6 mL/g VS	[108]
6	Wheat straw	1.2 mm	Reactor 2 L	167.8 mL/g VS	264.7 mL/g VS	[108]
7	Barley Straw	5 mm	Glass reactor 2 L	240 mL/g VS	370 mL/g VS	[112]
8	Waste activated sludge	Ultrasonic pretreatment	Semi-continuous reactors (15 days)		49% increase	[113]
9	Wheat straw	High hydrostatic pressure		31.8 mL	77.9 mL	[110]
10	Hyacinthus spp.	Microwave		137.18 mL/g-sub	221 mL/g-sub	[114]
11	Groundnut shell	2 mm	Batch		147.6 l_N_/kgFM	[28]
12	Groundnut shell	4 mm	Batch		180.7 l_N_/kgFM	[28]
13	Groundnut shell	6 mm	Batch		177.3 l_N_/kgFM	[28]

*Y*_BP_ = Yield before pretreatment and *Y*_AP_ = Yield after pretreatment

#### Chemical pretreatments

Chemical pretreatment is one of the pretreatment method that is more popular than physical and biological methods due to its effectiveness and the ability to improve biodigestion of complex feedstocks [115]. Hydrochloric acid (HCl), potassium hydroxide (KOH), sulfuric acid (H_2_SO_4_), lime (Ca(OH)_2_), aqueous ammonia (NH_3_∙H_2_O), sodium hydroxide (NaOH), acetic acid (CH_3_COOH), and hydrogen peroxide (H_2_O_2_) are some of the chemicals that has been investigated for the pretreatment of lignocellulose materials before anaerobic digestion.

##### Acidic pretreatments

One of the most popular pretreatment methods for lignocelluloses that were study widely is acidic pretreatment. Either dilute or strong acid (H_2_SO_4_, HCl or HNO_3_), pretreatment have been examined at high temperatures, and combination with other treatment like steam explosion has been examined also [116]. Lignin and hemicelluloses get solubilized when acid with strong concentration is used, but there is need to recover the acid. Lignin are redistributed and not solubilized when dilute acid concentration is used, and to achieve the neutral pH, neutralization before anaerobic digestion is very important [117]. Despite the fact that acid pretreatment is the commonest conventional pretreatment technique used in lignocellulosic feedstock treatment, and the generation of inhibitory materials like phenolic acids, furfurals, aldehydes, and 5-hydroxymethylfurfural make it less alluring. Because of the corrosive and toxic properties of the most acids, there is need to construct the digester that can withstand these characteristics [118]. Rice straw was pretreated with two levels procedures of dilute H_2_SO_4_ and aqueous ammonia in percolation mode and the reducing sugar produced was reported to be 90.8 and 96.9%, respectively, showing that lignin and hemicelluloses removal can be improve with the combination of the two processes [119]. Wheat and rice straw pretreated with acid technique produced highest sugar contents of 565 and 287 mg/g respectively, without hydroxymethyl furfural and furfural production [119, 120]. Oxalic acid was employed to pretreat corn cobs [121], the feedstock was heated to a temperature of 168 °C for 26 min and a cumulative sugar content of 13% was recorded, and low quantity of inhibitors was recorded. Marzialetti et al. [121] investigated the influence of various acids, viz., HCl, H_2_SO_4_, HNO_3_, TFA, and H_3_PO_4_ on loblolly pine in a batch digester. TFA produced the highest quantity of soluble monosaccharides at 150 °C and pH of 1.65. When newspaper was treated with acetic and nitric acid, 80% lignin removal was achieved [122] and rice straw pretreated with propionic and acetic acid increase the methane by 36% as against that of untreated rice straw [30].

##### Alkaline pretreatments

The use of Alkaline in lignin removal is very effective, but cellulose concentration remains at high level. Alkali pretreatment leads to fiber swelling which creates a larger surface area for accessibility; it reduces crystallinity and degrades the bond between lignin and carbohydrate which leads to the interruption of the lignin arrangement [123]. Wheat straw pretreated with alkaline pretreatment was noticed to release 100% methane yield increase [124]. 36–45% increase in methane yield was recorded from newspaper pretreated with alkaline subcritical water [125]. Pretreated rice straw with sodium hydroxide (4–10%) increased the methane yield by 3–58% [126], 112% increase was reported when 4% sodium hydroxide and hydrothermal pretreatment was combined together [127]. When calcium hydroxide was used to pretreat municipal solid waste, the methane yield was increased by 172% [128]. The quantity of catalyst used for the pretreatment and purchase price determines the cost of pretreatment; for instance, lime will cost less compare to sodium hydroxide, together with the expenses of recovery and further reuse [55]. Dahunsi et al*.* [129] noted that when sorghum bicolor stalk was treated with hydrogen peroxide, 73% and 42% of lignin and hemicellulose was removed, respectively, and cellulosic percentage was increased by 23%. The volume of biogas produced was increase by 65% when compared with the substrate pretreated with acid, and the retention period was reduced by 5 days. It was observed that aggressive dislocation characteristics of alkaline pretreatment can generate phenolic substances that can hindered the anaerobic fermentation of lignocellulosic material [130]. Alkali pretreatment method is economical, but the major disadvantage is its high cost at the downstream processing as the process needs large volume of water to remove the salts from the feedstock and removal process is an awkward process.

##### Oxidative pretreatments

The application of oxidizing agents such as ozone, FeCl_3_, hydrogen peroxide, and oxygen or air to solubilize the lignin and hemicellulose of lignocellulosic feedstock to enhance hydrolysis of cellulose is another chemical pretreatment technique [40, 123]. Oxidizing agent like hydrogen peroxide or per-acetic acid was dissolved in water and poured on biomass during oxidative pretreatment. The targets are partial breakdown of hemicelluloses and delignification of the biomass [131]. For wet oxidation method, oxygen is added into pretreatment digester at temperature of up to 200 °C and pressure of up to 1.5 MPa [31]. Earlier result was shown that at pH higher than 10, hydrogen peroxide addition was most efficient; but below this pH, no delignification was noticed. Success was also recorded when wheat straw was pretreated with alkaline peroxide [132]. Sweet sorghum bagasse was treated with various pretreatment techniques and the most yields were recorded from dilute NaOH and come next is H_2_O_2_ pretreatment. The optimum cellulose hydrolysis outputs were 74.3% and 90.9%, respectively, and cumulative sugar produced was 5.9, 9.5, and 19.1%, respectively, higher in comparison with the untreated experiment [133]. In this process, lignin are transformed to acids and caused delignification and may act as inhibitors, and this necessitated the removal of these acids formed [134]. Oxidative pretreatment techniques damage significant percentage of hemicellulose making them inaccessible for digestion and this is the principal challenge of the method [135].

##### Ozonolysis

Pretreatment of lignocellulosic materials with ozone is aimed at reducing lignin percentage as ozone majorly degrade only lignin and has no significant effects on cellulose and hemicellulose [101]. This method can be performed at ambient temperature and pressure unlike other chemical pretreatment methods. Likewise, it does not generate any toxic materials and is environment benign; and has no effect on other process like yeast fermentation and enzymatic hydrolysis after pretreatment [136]. When poplar sawdust was pretreated, percentage of lignin was reduced to 8% and the yield of sugar was increase to 57% [137]. Treatment of different types of feedstock with ozone method has shown a good result, and methane yield was improved by 66% when microalgae feedstock was pretreated with ozone [138]. Despite the effectiveness of this method, the high volume of ozone needed makes it uneconomical and not suitable for industrial scale pretreatment.

##### SPORL treatment

Sulfite pretreatment to subdue recalcitrance of lignocellulose (SPORL) is a novel and effective pretreatment technique for lignocellulose materials [139]. It can be accomplished in two stages; the feedstock is to be treated with either magnesium or calcium sulfite to get rid of lignin and hemicellulose contents in the first stage, while mechanical disk miller is utilized to shorten the size of the already pretreated feedstock significantly in the second stage. Spruce chip was pretreated with SPORL applying 8–10% bisulfite and 1.8–3.7% H_2_SO_4_ at 180 °C for the period of 30 min. At the end of 48 h of hydrolysis with 14.6 FPU cellulase + 22.5 CBU β-glucosidase per gram of substrate, it was reported that over 90% of the feedstock was transformed into cellulose [140]. Likewise, about 0.5% hydroxymethyl furfural (HMF) and 0.1% furfural (fermentation inhibitors) were produced when compared with 5% HMF and 2.5% furfural that was formed during acid catalyzed steam pretreatment of spruce. The quantity of HMF and furfural was also noticed to reduce with higher bisulfite. The feasible reason is that at the same acid charge, higher quantity of bisulfite results into higher pH which lowers the disintegration of sugars to HMF and furfural. Switch grass was pretreated with SPORL under the temperature ranges of 163–197 °C for the duration of 3–37 min with H_2_SO_4_ dosage of 0.8–4.2% and Na_2_SO_4_ dosage of 0.6–7.4%. The results showed an enhanced digestion of switchgrass by eliminating hemicellulose, while lignin dissolved partly and reduce hydrophobicity of lignin by sulfonation. Pretreated switchgrass was hydrolyzed by 83% within 48 h with 15 FPU cellulase and 30 CBU β-glucosidase/g cellulose [141]. Pretreatment with SPORL techniques reported to have yielded the optimum feedstock yield of 77.2% in comparison with dilute acid and alkali pretreatment that yielded 68.1 and 66.6%, respectively. Bagasse, corncob, water hyacinth, and rice husk were pretreated with sodium sulfide and sodium sulfite together with sodium hydroxide. At the optimum pretreatment conditions, the yields recorded were 75% lignin and 90% hemicellulose reduction from bagasse and corncob, while 97% lignin and 93% hemicellulose were eliminated from water hyacinth and rice husk [142]. SPORL pretreatment method has become familiar recently because of its effectiveness, variability, and easiness. It minimizes the energy intake to 1/10 needed for the biomass size reduction. The cellulose-to-glucose transformation rate is very high and high lignin removal and retrieval. It has the ability to process different biomass with superb scalability for commercial production by retrofitting into existent mills for biofuels production. Nevertheless, some important areas like large volume of water required for washing of the feedstock after pretreatment, sugar debasement, and high cost of recovery of the chemicals used during pretreatment have to be addressed to make SPORL an economical pretreatment method [143].

##### Organic solvent pretreatments

The use of organic solvents like methanol, ethanol, ethylene glycol, or acetone combined with or without inorganic catalyst to pretreat feedstock at high temperatures is called organosolv [144]. In this method, aqueous organic solvents were added to feedstock at a specific temperature and pressure [145, 146]. Generally, this process is performed with salt catalyst, alkali, or acid [143]. The type of feedstock and catalyst used determines the temperature of organosolv pretreatment and can be around 200 °C. The major focus of this method is to extract lignin that is a value-added product, while cellulose and hemicellulose syrup of sugars C5 and C6 are also released during organosolv treatment. In this pretreatment method, intra-molecular bonds are disrupted by organic solvent as a means to aid the breaking down of lignocelluloses by enzymes. There is a need for solvent recovery and reuse to some extent, and this can be accomplished in various extraction and filtration procedures, and to eschew inhibition during the anaerobic fermentation process, all the inhibitors must be removed from the substrate. Recovery method will determine the cost of the process, for instance evaporation and condensation; likewise the solvent cost [36]. Different factors like catalyst employed, duration of reaction, concentration of the solvent, and temperature will define the physical properties (fiber length, crystallinity, degree of cellulose polymerization, etc.) of the treated feedstock. Inhibitors to digestion are formed when the process temperature and acid concentration are high with long reaction time. H_2_SO_4_, NaOH, and MgSO_4_ were applied as catalysts for the treatment of pine, and H_2_SO_4_ was recorded as the most efficient catalyst with regards to ethanol yield, but for degradability, NaOH was reported to be most efficient when 2% concentration was used [147, 148]. H_2_SO_4_ appeared to be a very strong catalyst as a result of its high reactivity, but it is toxic and corrosive and generates inhibitory products. Exorbitant cost of solvents is the major disadvantage of this process, although reclaiming and recycling of the solvents by evaporation and condensation can reduce the cost. It is very important to remove the solvent, because it can have negative impacts on microorganisms’ growth, enzymatic hydrolysis, and digestion [149]. When compared with methanol, ethanol is less toxic and being the end-product makes it more acceptable as against other organic solvents [119, 120]. Notwithstanding, the ethanol presence will hinder the activities of hydrolytic enzymes and reduce the ethanol; therefore, water is utilized for enzymatic hydrolysis of hemicellulose and fermentation of pretreated feedstock [150]. Pretreatment of lignocelluloses with organic solvent *N*-methylmorpholine-*N*-oxide (NMMO) has been discovered to improve the methane yield greatly [54]. NMMO pretreatments are performed at comparatively low temperatures; more than 98% of the solvent can be retrieved without chemical derivatization and toxic waste pollutant is not produced. Portion of cattle and horse waste were treated with NMMO in the previous studies and there was 53 and 51% increase in methane yield, respectively [151], as well as on high-crystalline cellulose [152] and on an empty oil palm fruit bunch [153]. Ethanol was used successfully to pretreat sweet sorghum stalks before anaerobic digestion; the methane yield was increased by up to 270% in comparison with the untreated yield [144]. The use of alcohol-based organosolv pretreatment in conjunction with mechanical ball milling to pretreat Japanese cypress (*Chamaecyparis obtuse*) had been experimented; it was reported that ball milling of short time with alcohol-based organosolv treatment in bland conditions had a synergetic impact on the enzymatic digestion of the feedstock [154]. Poplar wood chips was pretreated with steam explosion and organosolv to separate lignin, cellulose, and hemicellulose of the materials, and it was noted that lignin extraction was improved by 66%; the two-stage pretreatment process retrieved 98% of cellulose, while 88% of the hemicellulose had been hydrolyzed to glucose after 72 h [155]. Organosolv pretreatment is less preferred due to the level of the risk required in the treatment of organic solvents that are highly flammable. If there is no assurance of adequate safety measures, it can lead to serious damage resulting to extensive fire detonations.

##### Carbon dioxide (CO_2_) explosion

This is a biomass pretreatment method that is carried out under supercritical CO_2_ when the gas acts like a solvent. This supercritical CO_2_ is discharged under a high-pressure vessel containing the feedstock [156]. The vessel and its content are heated to the needed temperature and maintained for some minutes at high temperatures [13]. CO_2_ is introduced to the feedstock at high pressure and releases carbonic acid that hydrolyzes the hemicellulose. When the pressurized gas is released, it breaks down the feedstock structure and improves the available surface area [157]. This method is not the best for feedstock with little or no moisture content, because hydrolytic process performs better when the moisture content is high [156]. The method is attractive because it required low temperature, minimal cost of carbon dioxide, non-formation of toxin, and high solid capacity. Notwithstanding, one of the principal challenges of the method is the high cost of reactor that can withstand higher pressure that is associated with the method, and this has hindered its usage in a commercial scale [149]. Another challenge of this method is the high thermal energy required for the disintegration of feedstock.

##### Ionic liquids

Ionic liquid application to pretreat lignocellulosic materials has gained special consideration in the last decade. They are relatively new category of solvents that are purely consisted of ions (anions and cations), with low melting points (< 100 °C), high thermal stabilities, insignificant vapor pressure, and high polarities [158, 159]. The mostly applied ionic liquids are imidazolium salts and they are presumed to contend with lignocellulosic constituent for hydrogen bonding thereby rupturing its network [160]. If appropriate anti-solvents can be selected, it is possible to achieve up to 80% hemicellulose degradation [161]. Avicel was treated with 1-butyl-3-methyl imidazolium chloride (Bmim-Cl) and 50% increase in enzymatic hydrolysis and twofold improvement in yield was recorded [162]. Wheat straw was also treated with Bmim-Cl and significant increase in enzymatic hydrolysis and yield was reported [163]. Furthermore, adding other treatments like chemical pretreatment is needed at times to neutralize the already treated feedstock and this can add to the cost of the production. It has been observed that it is not all pretreatments that were successful as the use of calcium hydroxide led to 14% decrease, but dry chemo-mechanical methods improve feedstock macro-porosity and increase microbial activities [164]. The method required specific and expensive equipment and high energy that can generate some inhibitors like 5-hydroxymethylfurfural (HMF) that can have adverse effects on succeeding digestion process [165]. Some recalcitrant compounds can be produced at liquid stage if it is carried out at temperature higher than 170 °C [166] which is another disadvantage of the process. Furthermore, treatment with additional chemical pretreatment technique is required at times, this is to neutralize the pretreated feedstock and this can add to the expenses of the process. Generally, irrespective of the feedstock used, methane yield was improved by 19–89% when pretreated with ionic liquid. Nonetheless, not all the chemical pretreatments were successful when combined with ionic liquid.

##### Ammonia fiber explosion (AFEX)/ammonia-based pretreatment

In this method, liquid ammonia is utilize to pretreat lignocellulose materials and it can also be called ammonia recycle percolation (ARP) or soaking aqueous ammonia (SAA). AFEX pretreatment method is carried out at ambient conditions, while ARP is carried out at high temperature [147]. SAA is the AFEX type that is performed with aqueous ammonia in a batch reactor at temperature ranges of 30–60 °C which reduces the liquid performance during pretreatment process [167]. During pretreatment with AFEX, lignocellulosic materials are heated with liquid ammonia (1:1) in an enclosed vessel at temperature between 60 and 90 °C and 3 MPa pressure for the duration of 30–60 min. After the temperature has been held for 5 min in the vessel, the vessel’s valve is released and the pressure is released explosively which leads to the ammonia escape and reduction in temperature of the system [168]. The process is similar to that of steam explosion, but ammonia is applied here instead of water. Lignocellulose materials pretreated with ammonia at high pressure and assigned temperature lead to swelling and structural shift in cellulose crystallinity and enhance the reactivity of the remaining carbohydrates after pretreatment. The arrangement of lignin is altered and increases the permeability and digestibility. This method does not release inhibitors like other pretreatments and this is highly required for downstream processing. The overall cost required to set up the process is minimal, because there is no need for additional stages such as detoxification, water washing, retrieval, and reuse of large volumes of water. When operated at the optimum conditions of ammonia loading rate, pretreatment duration, temperature, pressure, and moisture content, over 90% of celluloses and hemicelluloses can be transformed into fermentable sugars [169]. The ammonia used in this process can be recovered and reuse to reduce the total cost of the pretreatment process. Ammonia recycle percolation (ARP) process also uses ammonia during the process, and aqueous ammonia (5–15 wt%) is circulated through a reactor containing the feedstock. The required temperature range is 140–210 °C with reaction period of 90 min at percolation rate of 5 mL/min after which ammonia is recycled [46, 170]. ARP has the capacity to solubilize hemicellulose, while the cellulose remains unaltered [134]. The major disadvantage of ARP is the high energy required to hold on to the process temperature. Agricultural residues, herbaceous plants, and municipal solid waste have been experimented with AFEX and ARP and the result was satisfactory, while for hardwoods, ARP treatments has been found to be more effective [119]. Soaking aqueous ammonia (SAA) which is another technology that is using ammonia requires lower energy as it is carried out at low temperature of 30–70 °C.

##### Deep eutectic solvents

There are some solvents that have many properties related to that of ionic liquids that are relatively new. A deep eutectic solvent (DES) is a fluid that usually consists of two or three cheap and harmless constituents that have the ability of self-affiliation, mostly through hydrogen-bond interactions. They form eutectic composition that have lesser melting point in comparison to that of their separate components [171]. These DES have the ability to solve some of the major concerns related to ionic liquids and can be represented by the universal expression below

$${\text{Cat}}^{ + } {{X}}^{ - } {{zY,}}$$

where Cat^+^ is mostly any ammonium, sulfonium, or phosphonium cation, and *X* is a Lewis base, usually a halide anion. The complex anionic species are generated between *X*^−^ and either a Lewis or Bronsted acid *Y* (*z* is the number of *Y* molecules that interact with the anion) [172]. Majority of the DESs have utilized choline chloride (ChCl) as hydrogen-bond acceptor. ChCl is economical, biodegradable, and harmless ammonium salt that can be generated from biomass and has the ability to synthesis DESs with hydrogen donors like carboxylic acids, asurea, and polyols. DES resembles ionic liquids in terms of physical characteristics and behavior, but it differs from ionic liquids, because their compositions are not completely ionic and can be generated from non-ionic materials [171].

##### Natural deep eutectic solvents

A good number of natural products have brought into ionic liquids and deep eutectic solvents recently. Urea, amino acids, choline, sugars, and some other organic compounds belong to this category [173]. These solvents can be generated from natural sources and are referred to as Natural Deep Eutectic Solvents (NADES). NADES are economical, non-toxic, easy to synthesize, highly biodegradable, and biocompatible unlike ionic liquids. In addition, some studies have shown that these solvents can be retrieved and reused with high efficiency. NADES made by the complex formation that exists between hydrogen acceptor and hydrogen-bond donor. As a result of the charge delocalization of the individuals’ components, the melting point of the prepared solvent is usually low. After examined the potential of NADES in various applications, these solvents have been identified as the twenty-first century solvent [174]. Furthermore, recent research on the pretreatment of lignocellulose materials with NADES reagents established its high specificity toward lignin solubilization and extraction of high purity lignin from agricultural residues like rice straw [175]. In spite of having substantial ability for the extraction of natural products, being solvents with high viscosity is its demerit. The dilution effect of physicochemical characteristics of NADES was investigated by Dai et al. [173], and FT-IR and HNMR showed high energetic H-bonds between the two mixtures of NADES system. Nevertheless, the interactions are weakened when it is diluted with water, when diluted with about 50% (v/v) with water, and the hydrogen interactions vanished totally. NADES viscosity lowered to the order of water and conductivity rise up to about 100 times for some NADES reagents. When compared with other pretreatment techniques, NADES can be the game changer in concept in enzyme, food processing, and pharmaceutical industries. It has been presumed that the use of dry chemo-mechanical pretreatment techniques will improve the feedstock macro-porosity and encourage microbial xylanase activity [164].

The impacts of various chemical pretreatments on some feedstock are presented in Table 3. The table shows that the effects of these pretreatment are not the same despite being under the same chemical pretreatment methods. In the same vein, it can be deduced that when different methods were applied to the same feedstock, and the results are not the same. Corn straw was pretreated with HCl (2% v/v), NaOH (8% v/v), and NH_3_ (10% v/v), it was reported that biogas yield from treatment with HCl, NaOH, and NH_3_ was increased by 216.7, 62.9, and 67.7 mL/g VS, respectively [176]. This difference in yield increase can be researched further with the same concentration of chemical used to be able to establish the best chemical for the feedstock in use, likewise the chemical concentration that can give the yield increase similar to that of HCl needs to be established. In the case of sorghum bicolor stalk, H_2_SO_4_ and H_2_O_2_ were used as pretreatment and it was reported that hemicellulose was degraded by 10.5 and 17.5%, while lignin were degraded by 41.8 and 9.2%, respectively [129]. This results shows that H_2_O_2_ has ability to degrade hemicellulose more than H_2_SO_4_, and for lignin degradation, H_2_SO_4_ is the more preferred acid for sorghum bicolor stalk. In terms of methane yield, H_2_SO_4_ improved by 6.5% when compared with untreated yield, whereas H_2_O_2_ reduced the methane released by 0.5% when compared with the untreated feedstock [129]. This shows that the expected end-products will determine the choice of chemical pretreatment to be applied.

**Table 3:** Different chemical pretreatment applied to the biogas production and yield

S/N	Biomass	Pretreatment	Degradation of hemicellulose (%)	Degradation of lignin (%)	Anaerobic digestion condition	*Y*_BP_	*Y*_AP_	Refs
1	*Agave tequilana* bagasse	2% (w/w) HCl			Batch at 32 °C, pH 5		0.26 L CH_4_/g COD	[177]
2	Pinewood	Organosolv (150 °C, 1 h)	21.3	26.5	250 mL conical flask at 39 ± 1 °C	38.7 ± 4.1 CH_4_ (L/kg CH)	63.3 ± 9.3 CH_4_ (L/kg CH)	[178]
3	Sugarcane bagasse	Alkaline autoclaving			Batch at 35 °C for 30 days	222 mL CH_4_/g VS	420 mL CH_4_/g VS	[179]
4	Wheat straw	Urea (1% w/w)			Batch at 35 °C, 120 rpm	210.4 mL CH_4_/g VS	305.5 mL CH_4_/g VS	[180]
5	Corn Straw	Hydrochloric acid 2% v/v			1 L Erlenmeyer flask at 37 ± 1 °C	100.6 mL/g VS	216.7 mL/g VS	[176]
6	Corn Straw	Sodium hydroxide 8% v/v			1 L Erlenmeyer flask at 37 ± 1 °C	100.6 mL/g VS	163.5 mL/g VS	[176]
7	Corn Straw	Ammonia 10% v/v			1 L Erlenmeyer flask at 37 ± 1 °C	100.6 mL/g VS	168.3 mL/g VS	[176]
8	Cotton gin waste	Ethanol 0.5 mmol/g VS substrate			250 mL conical flasks at 35 °C	172.5 mL/g VS	241.5 mL/g VS	[181]
9	Sorghum bicolor stalk	H_2_SO_4_	10.5	41.8	250 mL batch reactors at 37 °C	55% CH_4_	54.5% CH_4_	[129]
10	Sorghum bicolor stalk	H_2_O_2_	17.5	9.2	250 mL batch reactors at 37 °C	55% CH_4_	61.5 CH_4_	[129]

#### Nanoparticles’ pretreatment

In the past few years, interdisciplinary research in the nanostructure science and technology has gained explosive attention globally. It was observed to have the capacity to revolutionizing the arrangement of materials and products; and improve their accessibility. With the recent breakthrough in nanotechnology, it is expected to supply wide range of nano-materials that can be utilized for enzymes’ immobilization. It is generally agreed that enzymes’ immobilization on nanoscale materials is an uncommon and bright method to improve the catalytic efficient of enzyme [31, 182, 183]. The nano-particles that immobilized enzymes are referred to as ‘nanobiocatalyst’. The real enzyme immobilization with the use of cross-linking molecules gives a spacer that reduces stiff impediment between enzyme and the solid base resulting in improvement in the flexibleness of immobilized enzymes [184]. Research has shown that many nano-particles (NPs) can absorbed and/or reacted with membranes of the cell and break them. Nanomaterials have the ability to improve the effectiveness of immobilized enzyme, since they provide enough surface area for the enzyme attachment and this enhances enzyme loading per unit mass of particles [185, 186]. The use of nanobiocatalysts in the pretreatment of lignocellulosic materials before hydrolysis has a bright assurance to turn around the whole scenario [187]. Zhang et al. [188] reported that ZnO NPs destroyed the bacterial cell membrane, while Ma et al. [189] opined that the cell membrane disruption and/or cell dead was as a result of physical pierce of CeO_2_ NPs and the oxidizing ability of dissolved Ce4^+^ on the outward membranes of microorganisms in an anaerobic digestion process. It has been reported that some NPs can influence strong inhibitory products during anaerobic digestion. For instance, NPs that composed of CuO, Mn_2_O_3_, ZnO, Cr_2_O_3_, CO_3_O_4_, CoO, and Ni_2_O_3_ have been reported to release visible growth inhibitory effects that were noticed to be connected to membrane destruction and oxidative stress responses [190]. Strong Ag NPs was reported to hinder the respiration of nitrifying organisms at 86.3% inhibition rate [191] and it was also observed that ZnO NPs with higher dosages hindered the hydrolysis, acidification, and methanogenesis stages during the anaerobic fermentation of sludge. The application of Fe NPs was reported to efficiently minimize the percentage of H_2_S in biogas produced and improved methane yields in some cases [192]. Micro-sized and nano-sized CuO (5–30 mm) and ZnO (15 mm, 50–70 mm) were used to pretreat cattle manure before digestion for 14 days at 36.1 °C and nano-sized CuO and ZnO produced negative result, and this can be traced to the toxic behavior of nano-materials on the bacteria, 15 mg/L nano-sized CuO reduced biogas yield by 30%, while micro-sized CuO had no significant effect [193]. TiO_2_ NPs (1120 mg/L, 7.5 nm) were used to treat waste sludge prior to anaerobic digestion for 50 days and digested at both mesophilic (37.1 °C) and thermophilic temperature (55.1 °C), the result showed 10% improvement in biogas yield [194], and 11% increase in biogas was recorded when 10 mg/L of CeO_2_ was utilized. When an anaerobic digestion was exposed to Fe NPs, the percentage of H_2_S was reduced significantly and improved methane release [192]. The use of magnetic acid-functionalized nano-particles can combine the merits of acidic catalyst with the merits of being retrievable and reusable together.

Direct interspecies electron transfer (DIET) through Fe_2_O_3_ NPs can also play a crucial part in enhancing the methanogenesis process of anaerobic digestion. Basically, the reduced electron carriers are expected to transform into CO_2_. This pattern of syntrophy and methanogen electron interchange is regarded as interspecies electron transfer (IET) [195, 196]*.* In DIET, available materials that occurred naturally or artificially created can be employed for electron transfer. Fe_2_O_3_ NPs that are semi-conductive mineral type can act as electron conduits between the electron donors and acceptors thereby expedite the release of methane from the reduced electron carriers and CO_2_ [196]. The NPs in this process are related to the enzymes in the catalytic reactions in a sequence of biochemical reactions [197]. The unequaled characteristics of nano-particles were employed by Casals et al. [198] and utilized iron-oxide NPs that dissolve slowly to release the living microorganisms with the needed iron ions without any toxicity to the bacteria. When Fe_3_O_4_ NPs (7 nm) with a concentration of 100 ppm was added to anaerobic reactor set-up at mesophilic temperature (37 °C) for hydraulic retention period of 60 days, the result showed 180 and 234% improvement in biogas and methane released, respectively, and this was regarded as the best improvement to biogas generation utilizing NPs by the authors [201]. Comprehensive application of nano-particles in industrial and consumer products has caused concerns about their potential impacts on the environment; therefore, the impacts of various NPs (Ag, MgO, Fe_2_O_3,_ and nZVI [nano zero-valent iron]) on the anaerobic fermentation of lignocellulose materials have not been investigated deeply.

The biogas and methane yield from different nanoparticle pretreated feedstock is presented in Table 4. It can be observed that different nano-materials were found to be suitable for different feedstock. Waste activated sludge was pretreated with TiO_2_, Ag, Fe_2_O_3_, and ZnO, and the result shows that all of them have different effect of the same feedstock [199–202]. Another important factor that is noticed to have indicative effect on nanoparticle pretreatment is the particle size of the nanomaterial and its concentration. In the case of pretreatment of sludge from UASB with 850 mm particle size of ZnO with two different concentrations of 10 mg/L and 1000 mg/L. The result showed that the biogas yield was decreased by 8% for 10 mg/L, while it was 65% decrease in biogas yield when the concentration was 1000 mg/L [203]. This result shows that the higher the concentration of the nanoparticle, the lower the biogas yields. Likewise, when the same concentration (10 mg/L) of CeO_2_ and ZnO was used to pretreat sludge from UASB, the results showed that biogas yield was improved by 11 and 8% for CeO_2_ and ZnO, respectively [203]. This indicates that different nano-materials have different concentration for optimum yield. Different behavior was reported for different types of NPs during biological pretreatment processes; therefore, there is need to establish new guidelines for the application of various NPs to enhance the anaerobic fermentation of sludge and reduce the inhibitory materials. In the case of lignocellulose biomass, there is a limited literature with regards to nanoparticle pretreatment and this necessitated the urgent needs to encourage more research in this particular pretreatment technique that has been utilized and adjudged to be satisfactory in sludge pretreatment.

**Table 4:** Different nano-particles pretreatment applied to the biogas production and yield

S/N	Biomass	Pretreatment	Size	Concentration	Anaerobic digestion condition	Effect	Refs
1	Fresh raw manure	Fe_3_O_4_	7 nm	20 mg/L	37 °C for 40 Days	73% and 115.66% increase in biogas and methane yield, respectively	[204]
2	Sludge from UASB reactor	CeO_2_	192 nm	10 mg/L	30 °C for 40 Days	11% Increase in biogas production	[203]
3	Fresh raw manure	Co	28 nm	1 mg/L	37 °C for 40 Days	71% and 45.92% improvement in biogas and methane released, respectively	[204]
4	WAS	ZnO	140 nm	10 mg/g-TSS	35 °C for 40 Days	No effect	[202]
5	WAS	Fe_2_O_3_	< 30 nm	100 mg/g-TSS	37 °C for 40 Days	117% increase in methane production	[201]
6	AGS	CuO	37 nm	1.4 mg/L	30 °C for 83 Days	15% Decrease in methane production	[205]
7	Waste sludge	Ag	40 nm	184, 77 and 6.3 mg/kg	36 °C for 38 Days	No effect	[200]
8	WAS	TiO_2_	185 nm	150 mg/g-TSS	35 °C for 105 Days	No effect	[199]
9	Sludge from UASB	ZnO	850 nm	10 mg/l	30 °C for 40 Days	8% Decrease in biogas production	[203]
10	Sludge from UASB	ZnO	850 nm	1000 mg/l	30 °C for 40 Days	65% Decrease in biogas production	[203]

#### Thermal pretreatments

Thermal pretreatment method is technique whereby the lignocellulosic feedstocks are heated at high temperature. At high temperature of 150–180 °C, lignin and hemicelluloses begin to solubilize, and their composition and arrangement are determined by the branching groups of the hemicellulose. There are different techniques by which this heat pretreatment can be applied to the lignocellulose feedstock.

##### Liquid hot water

This technique is also referred to as hot compressed water and is like steam pretreatment technique as the name insinuate, water at very high temperature (170–230 °C), and pressure (up to 5 MPa) is applied rather than steam. It hydrolyzes hemicellulose and gets rid of lignin and making cellulose more available while inhibitors at high temperature are avoided [206]. In decades, pulp industries have been using hot water as pretreatment for lignocelluloses [51]. Liquid hot water pretreatment can be applied in three manners: counter current, co-current, and flow-through pretreatment. It was reported that at temperatures between 200 and 210 °C, methane produced was reduced and this can be linked to possible production of refractory materials [207]. Rice straw, Japanese cedar, Nipa frond, and Japanese beech were pretreated with two-step hydrolysis (step I: 230 °C–10 MPa–15 min; step II: 275 °C–10 MPa–15 min), and they were reported to solubilize at 97.9, 82.3, 92.4, and 92.2% respectively [208, 209]. It has been reported that liquid hot water has the ability to pretreat a good number of feedstock and softwoods inclusive [210]; nevertheless, energy required at the downstream processing is high because of large volume of water required [149].

##### Steam explosion

In steam explosion method, feedstock are subjected to steam at specific temperature and pressure, and it was suggested to be economical pretreatment method for debasement of lignocellulosic materials, but at times, xylan portion is partially debased and the process can produced inhibitors [211]. In steam explosion method, already coarsely shredded or chipped lignocellulosic materials are put in a pressure vessel containing high-pressure steam. The duration of treatment can be from 30 s to 30 min, under a temperature of between 120 and 260 °C, and pressure ranges between 5 and 20 bars. When the pretreatment time lapse, the substrate will be transferred into a flash tank under atmospheric pressure and this will cause immediate decompression and deconstruction of the substrate. Substrate that belongs to acetyl groups releases organic acids during steam explosion process and this can act as catalyst during the hydrolysis process. The hemicellulose and lignin get degraded or solubilized due to these acids that catalyzed the hydrolysis reaction [55].

To improve the steam explosion pretreatment the more, acid or alkali can be added during the process. About 20% improvement in methane released was recorded when wheat straw pretreated with steam explosion is examined with control [212]. Moisture content of the feedstock, particle size, temperature, and treatment time are that factors that affect steam pretreatment [210]. It was reported that low temperature and higher treatment duration (190 °C for 10 min) produced preferable result in comparison with higher temperature and lesser treatment duration (270 °C for 1 min), because less digestion inhibitory product formed in the previous treatment time. Previous studies have shown that 67% increase in methane produced was recorded when bio-fibers from digested manure was pretreated with steam explosion as against untreated ones [213]. On bulrush, a methane liberation of 205 mL/g VS was recorded when treated with steam explosion [214], while for salix, 240 mL/g VS [215] was recorded. When compared with milling for size reduction, steam explosion required little energy and the technique has been considered as the most cost efficient [46]. Steam explosion at temperature of 180 °C was used to pretreat sugarcane bagasse using 1.0% acetic acid and 17.45 min reaction time before anaerobic digestion. The biogas yield of 434.47 L/kg VS which was 91.88% higher than the unpretreated feedstock yield of 2226.42 L/kg VS [216]. Steam pretreatment has been adjudged to be efficient for the pretreatment of residues from agriculture and hardwood, although, for efficient pretreatment of soft woods, acid catalyst needs to be added. Some of the benefits of steam pretreatment technique are: energy required is low, chemicals required is limited, no cost of recycling needed, and environmental benign. The tendency to form digestion inhibitors at higher temperature, partial digestion of lignin–carbohydrate matrix, and the demand to remove the hydrolysate which reduce the sugar produced by 20% are some of the drawbacks of steam pretreatment method [149].

##### Wet oxidation

Simple treatment of lignocellulosic materials with air/oxygen together with hydrogen peroxide or water at temperature beyond 120 °C for 30 min is called wet oxidation [217]. This technique has been employed to treat waste-water and soil redress, and it was reported to be suitable for feedstock enriched in lignin [218]. Effectiveness of wet oxidation relies on temperature, oxygen pressure, and reaction time. At temperature above 170 °C, water acts like acid and catalyzes hydrolytic processes in this method. Lignin experiences oxidation and hemicelluloses are disintegrating into smaller pentose monomers, while celluloses partially affected by this method. It has been reported that the application of chemical agents such as sodium carbonate and alkaline peroxide to the process increased hemicellulose debasement, lessens the reaction temperature, and minimizes the production of inhibitory substances like furfurals and furfuraldehydes [219]. Wet oxidation pretreatment was applied to common reed (*Phragmites australis*) and the digestibility was increased by threefold, and hemicellulose and lignin were solubilized by 51.7 and 58.3%, respectively, while 82.4% of the cellulose was transformed during enzymatic hydrolysis [220]. Rice husk was pretreated with wet oxidation at temperature of 185 °C and 0.5 MPa pressure for 15 min and 67% cellulose removal recorded, 89% lignin removed, and 70% hemicellulose solubilized [221]. When alkaline peroxide-supported wet air oxidation was used to pretreat rice husk, 88 and 67 wt% of lignin and hemicellulose were solubilized, respectively [219]. This technology is not encouraged at industrial level because of the natural combustion nature of pure oxygen and exorbitant cost of hydrogen peroxide [143].

##### Hydrothermal

Of recent, attention has been shifted to hydrothermal pretreatment because of its effectiveness in biomass penetration, hemicellulose removal, hydration of cellulose, and partial lignin removal. The technique does not require chemicals and materials that have high resistant to corrosion, and these are important benefits of the method [127]. Distinctively, this method can remove larger percentage of the hemicellulose and certain percentage of lignin in lignocellulose materials by degrading them into soluble fractions and also alienate the recalcitrant arrangement [222]. The major important factor that influences this method is temperature [223]. Usually, hydrothermal pretreatment is carried out at temperature range of 90–260 °C [224]. Napier grass was pretreated with hydrothermal at 175 °C for 15 min reaction time, and 25% improvement in methane released was recorded when compared with untreated feedstock yield [225]. It was also noticed that the formation of inhibitors like 5-hydroxymethylfural and furfural that affected the methanogenesis was recorded at 200 °C. In a similar research, wheat straw was pretreated with hydrothermal under different temperatures of 120, 140, 160, and 180 °C, optimum yield which was 53% increase in methane released when compared with untreated wheat straw was recorded when the temperature was 180 °C [226]. Safflower straw was pretreated at 120, 150, and 180 °C for 1, 2, and 5 h, and the highest methane yield was recorded at the minimum severe conditions (120 °C for 1 h.) with 98.3% increase with regards to untreated straw [227]. In another research, rice straw was pretreated with hydrothermal technique at temperature ranges from 90 to 130 °C, and the methane yield from 100 to 130 °C was similar (127.6 and 124.6 mL/g VS, respectively) and they were 22.9 and 19.83%, respectively, better than untreated rice straw [228]. Antwi et al. [229] investigated the effect of hydrothermal pretreatment on the residue of cocoa pod at temperature ranges from 155 to 220 °C for the period of 15 min. The highest methane yield of 526.38 mL/g VS was recorded at 150 °C for 15 min and also noticed that higher process conditions lead to lesser biogas yield.

Table 5 shows the effect of thermal pretreatments on some lignocellulose materials. It can be observed that temperature and process time is an important factor in this method. Reed biomass pretreated with steam explosion at different temperature and time produced different results. Biogas yield increase of 38 mL/g VS was recorded in steam explosion when temperature was 160 °C for 5 min, while 167 mL/g VS yield was recorded when temperature was 200 °C for 15 min [230]. In this case, higher temperature with almost the same treatment period leads to higher margin of biogas yield. However, in the case of conventional heating of wheat straw, lower temperature with lower treatment period produces the better biogas yield. When wheat straw was pretreated with conventional heating at 121 °C for 60 min, the biogas released was increased by 29% [112], and when another author pretreated the same wheat straw with conventional heating at 120 °C for 30 min, the biogas yield was improved by 64.3% [112]. The optimum pretreatment condition (conventional heating at 120 °C for 30 min) that produce the better result in wheat straw was applied to maize stalk, but it has no effect [112]. There is need to have a standard where the optimum temperature and time for different thermal pretreatment methods for various lignocellulosic materials will be listed, so that researchers and industries can have the needed information with ease, since different materials respond to different temperature and time.

**Table 5:** Different thermal pretreatment applied to the biogas production and yield

S/N	Biomass	Pretreatment	Anaerobic digestion condition	*Y*_BP_	*Y*_AP_	Refs
1	Corn stover	Steam explosion	Batch, 37 °C for 28 days	155.4 mL CH_4_/g VS	217.5 mL CH_4_/g VS	[231]
2	Rice straw	Hydrothermal 100 °C; 10 min	Glass Bottle 1 L	92 mL/g VS	280 mL/g VS	[228]
3	Reed biomass	Steam explosion 160 °C; 5 min	Digester 0.25 L	188 mL/g VS	226 mL/g VS	[230]
4	Reed biomass	Steam explosion 200 °C; 15 min	Digester 0.25 L	188 mL/g VS	355 mL/g VS	[230]
5	Safflower straw	Hydrothermal 120 °C; 60 min	Glass bottle 0.118 L	96.5 mL/g VS	191.4 mL/g VS	[227]
6	Cocoa pods residues	Hydrothermal 150 °C	Bottle 0.5 L	196.3 mL/g VS	289.3 mL/g VS	[229]
7	Wheat straw	Conventional heating (121 °C for 60 min)	Batch mesophilic		29% increase	[179]
8	Wheat straw	Conventional heating (120 °C for 30 min)	Batch mesophilic		64.3% increase	[112]
9	Maize stalks	Conventional heating (120 °C for 30 min)	Batch mesophilic		No change	[112]
10	Giant reed	Liquid hot water (190 °C for 15 min)	Batch mesophilic		+ 31% vs. 0 mL CH_4_/g control	[232]

#### Biological pretreatments

Biological pretreatment is an alternative method that is more eco-friendly and required less energy input [143]. This method uses only microorganisms, enzymes, or consortia to enhance the biodegradation of lignocellulosic materials thereby improve the biogas yields. The microorganism introduced in this method degrades the lignin content of the feedstock for biogas production. When compared with chemical and mechanical pretreatment methods, it is assumed to be the most effective, environmental friendly, and least-energy technique. There are several cellulolytic and hemicellulolytic microbes in the nature that can be particularly aimed for effective pretreatment of biomass [233]. Microorganism like white, brown, and soft-rot fungi that majorly debase lignin and hemicellulose and certain percentage of cellulose can be used for biological pretreatment [234]. An example is the use of fungi (both white-rot and brown-rot), enzymes, ensiling, and bacteria to degrade the biogas feed stocks. Biological pretreatment method required little energy consumption and does not require chemicals, and these are advantages of the method. Nevertheless, degradation of cellulose and hemicellulose together with the lignin as a result of lengthy incubation period is the drawback of the method [235].

##### Fungi

Fungi pretreatment is a method that required low energy and chemical, and minimizes the release of unwanted products [236]. White, brown, and soft-rot fungi are utilized for this process. White-rot fungi are capable of producing enzymes that has high hydrolytic ability to degrade lignocelluloses like lignin peroxidase, lacasse, and manganese peroxidase. Lignin degradation by white-rot fungi occurs as a result of lacasses and peroxidases (lignin-degrading enzymes) present in it [101]. Lignin destructive fungi have been used mainly for biological pretreatment. A cluster of basidiomycetes known as whit-rot fungi are recognized to first breakdown the lignin, while majority of the cellulose and hemicellulose remain unaltered. Ligninolytic enzymes such as laccase, lignin peroxidase, and manganese peroxidase were excreted by these fungi [235]. During fungi pretreatment, feed stock, and fungi were inoculated at room temperature for some weeks. White-rot and brown-rot fungi were used to pretreat rice straw and the methane released were increased by 46 and 31%, respectively [236]. In another research on olive mill waste-water, white-rot fungus was used to remove phenolic compounds and the process released an improved biogas [237]. *Pleurotus ostreatus* and *Trichoderma reesei* were employed to enhance the biodigestion of rice straw; at moisture of 75%, *P. ostreatus* was most efficient and attained 33.4% of lignin destruction and methane produced was increased up to 120% when compared with control [238]. Nevertheless, long duration of processing (about 30 days), accurate situations of growth, and reduction in organic matter due to microbial activities are some of the disadvantages of this method.

##### Enzymes

Enzyme pretreatment is a quick technique that can be performed within few hours, because the enzymes are smaller compared to microorganisms. Likewise, enzymes hold excellent mobility, solubility, and utmost relationship with the feedstock [239]. The cellulose biological hydrolysis is conducted with enzymes that have endogluconase, exogluconase, and β-glucosidase properties, while hydrolysis of hemicelluloses needs a good number of enzymes which includes endo-xylanase, α-glucuronidase, endo-mannanase, etc. [240]. Significant production of total phenolic compounds (TPC) was recorded in this method when willow was utilized; TPC levels of up to 195 mg/L were recorded in liquid portion and no inhibitory levels were recorded during the anaerobic fermentation process [241]. It was also reported that the maximum TPC values belong to the feedstock with higher lignin percentage. The experiment also recorded 24% improvement in methane produced from corn stover when enzymatic pretreatment was applied to pretreat corn stover. In another related experiment, methane released from corn stover pretreated with blend of cellulase enzymes was increased by 111% [242]. Conjunction of laccase and steam explosion as pretreatment before anaerobic digestion of bio-fibers has yielded a positive result [213]. Nevertheless, when bio-fibers were pretreated exclusively with lacasse, there was no improvement. As alluring as this methods is, at times it needs other pretreatments techniques like sterilization and the high cost of enzymes are some of the challenges facing the economic reality of it at the industrial level [243, 244].

##### Ensiling

Ensiling is a pretreatment method that is usually utilized to store wet feedstock prior to processing [245]. In this method, soluble carbohydrates are converted to acetic, butyric, lactic, and propionic acid by microorganisms. The pH is lower to below 4 during this process and this hinders the microorganisms’ growth, while the transformation of feedstock is favored [21]. Franco et al. [245] opined that ensiling can increase methane yield under particular conditions. Methane produced from residues of amaranths ensiled was significantly improved by up to 31% when compared with the ones without ensiling [246]. When corn stover was ensiled, the biogas produced daily and methane produced were reported to be twice the quantity of the one produced by control [247]. Lignocellulosic feedstock type, particle size, humidity, environmental conditions, etc. are some of the factors that determine the production performance of this method. Another advantage of the method is that feedstock will be available all year round without waiting for cultivation periods, but if the silage is not handle properly, it can lead to the loss of about 40% of its methane.

##### Bacteria

Bacterial with high hydrolytic ability has been employed for biological pretreatment. *Pseudomonas, Escherichia coli, Salmonella*, etc. that have the ability to synthesize celluloses have been observed by some studies [248, 249]. Methane released was improved by up to 158.7% when nine bacterial strains that have endoglucanase properties were used for biodegradation of microalgae [250]. An increase of 38% of biogas produced was recorded when a microbial consortium with high cellulolytic activeness known as MCHCA was employed for biodigestion of maize silage [251].

##### Microaerobic pretreatment

Microaerobic pretreatment (MP) has been investigated as an alternative treatment for corn straw by different researchers. It has been reported recently that supplying limited quantity of oxygen (or air) into the anaerobic digestion directly or during pretreatment process can enhance the methane yield of corn straw. The definite availability of phylum *Firmicutes*, class *Clostridia,* and order *Clostridiales* that are connected to hydrolysis of anaerobic digestion were grown under microaerobic conditions. In addition, the definite availability of *Methanobacterium* and *Oxytolerant* were both doubled under microaerobic conditions. Improvement in anaerobic digestion process in this case can be traced to the shifting of the microbial community under microaerobic conditions [252]. The quantity of oxygen introduced during pretreatment is very crucial, because disproportionate oxygen hinders the activities of the methane-forming microorganisms and lowers the methane yield [253]. In contrast, exuberant oxygen can oxidize the useable feedstock easily or help aerobic *Methanotrophs* to deplete methane. It has been noticed that thermophilic microaerobic pretreatment (TMP) prior to anaerobic digestion of corn straw led to improvement in the relative availability of phylum *Firmicutes* that are connected to the liberation of extracellular enzymes. The relative availability of phylum *Firmicutes* (particularly class *Bacilli*, order *Bacillales*) was higher under microaerobic conditions than anaerobic conditions, which allows and improves in extra cellular enzymes, volatile fatty acids (VFAs), reducing sugar, and soluble chemical oxygen demand (SCOD) under microaerobic conditions. Hence, the anaerobic digestion of corn straw was more effective and methane yield was improved [254].

##### Bioaugmentation

Bioaugmentation is a process where certain exogenous microorganisms are introduced to microbial community. It can be utilized to infix particular microorganisms into the biogas digester directly to enhance specific stages of anaerobic digestion [255]. This technique is used to enhance the start-up of a digester [256], improve the process performance, or improve the degradation abilities of a consortium [257]. It is an effective technology that has different merits; it does not require prior pretreatment, thereby simplify the process and give opportunity to develop other processes that are economical [240]. In addition, bioaugmentation has been used to recover biogas digesters that have been malfunctioning as a result of volatile fatty acids accumulation or due to high load rates [258]. As regards the utilization of bioaugmentation to improve biogas production from lignocellulosic materials, microorganisms have been examined alone or in combination with other microorganisms with high lignocellulosic degradation abilities. Several studies have reported that bioaugmentation with cellulolytic bacteria or bacteria consortia can improve the hydrolysis rate and accordingly improve the methane yield from lignocellulose materials like wheat straw [255, 259, 260], cellulosic waste materials released from sweet corn processing [37], and cellulose from corn stover [261]. Biogas yield was increased by 47% when *Enterobacter ludwigii* was introduced compared to when *E. ludwigii* was not added [262]. Microorganisms like *Clostridium stercorarium* and *Bacteroides cellulosolvens* that have high lignocellulose properties have been utilized for consortium improvement and to improve degradability of cellulose, hemicelluloses, and lignin in combination with thermal pretreatment (100–150 °C), and debasement of 78.2, 89, and 33.7% were reached, respectively, while the methane yield was improved by 246% [263]. Martin-Ryals et al. [37] recorded that usual bioaugmentation with a cellulolytic culture for pretreatment of cellulosic materials in the acid phase during the two phases anaerobic digestion increased the liberation of methane by 15% when compared with one-time bioaugmentation. Nevertheless, Ács et al. [38] reported that during bioaugmentation, microbial community was increased when utilizing a single phase and this made it possible to achieve an increase in biogas yield. Methane yield was increased by 17% when brewery spent was bioaugmentated with *Pseudobutrivibrio xylanivoras* Mz5T [264]. Moreover, adding fermentative/hydrolytic bacteria leads to increase in hydrogen concentration that can encourage the growth of hydrogenotrophic methanogenesis leading to better methane yields [38, 261]. Though, some of these studies were carried out in a controlled environment, such that it is easy to guarantee the survival of exogenous microorganisms [265]. Sometimes, the contribution of exogenous microorganisms introduced to the process effectiveness is small because of their metabolic abilities that are not sufficient to change to the inherent bacterial population and are hereby flushed out of the process. Despite the ability of bioaugmentation for hydrolysis improvement and eventually improve the biogas release, their strength has not been sufficiently substantiated, because the degradable portions of carbohydrate are mostly protected by lignin in the original substrate, and thereby reducing their availability to enzymatic and microbial degradation. The percentage degradation of hemicellulose and lignin, biogas, and methane of some lignocellulose materials pretreated with different biological pretreatment methods is as shown in Table 6.

**Table 6:** Different biological pretreatment applied to the biogas production and yield

S/N	Biomass	Pretreatment	Degradation of hemicellulose (%)	Degradation of lignin	Anaerobic digestion condition	*Y*_BP_	*Y*_AP_	Refs
1	Corn stover	Enzymatic (laccase)	–	–	Batch at 35 °C for 30 days	191.7 mL CH_4_	238.4 mL CH_4_	[241]
2	Rice straw	Fungal	–	33.4	Batch at 37 °C for 20 days	127 mL CH_4_/g VS	263 mL CH_4_/g VS	[238]
3	Corn stover silage	Fungal	32.4	22.6	Batch at 37 °C for 30 days	215.5 mL CH_4_/g VS	265.1 mL CH_4_/g VS	[247]
4	Yard trimmings	Fungal	9.8–16.2	14.8–20.2	Batch at 37 °C for 28 days	20 mL CH_4_/g VS	34.9–44.6 mL CH_4_/g VS	[266]
5	Sawdust	Microbial consortium, 10 days			Digester 5 L	89.9 mL/g VS	155.2 mL/g VS	[267]
6	Rapeseed stems and leaves	Rumen fluid, 24 h			Reactor 1 L	485.5 mL/g VS	507.9 mL/g VS	[268]
7	Corn stover	Fungi, *Pleurotus* *eryngii*, 30 days			Bottle 0.25 L	301.5 mL/g VS	360.4 mL/g VS	[269]
8		*Clostridium stercorarium* (100–150 °C)	89	33.7			246% increase	[263]
9	Marine macroalgae	β-glucosidase, pectinase, and carboxy-methyl-cellulase (50 °C for 2 h 100 rpm)			Batch 37 °C		54.6% increase	[270]
10	Corn stover	Laccase (30 °C for 24 h.)			Batch mesophilic		25% increase	[241]
11	Cotton stalk	Thermophilic microbial consortium (50 °C for 8 days)			Batch mesophilic		136% increase	[271]
12	Wheat straw	Bacteroidetes (37 °C for 15 days)			Batch mesophilic		80.3% increase	[272]
13	Corn straw	Microaeration (5 mL O_2_/g VS at 55 °C)			Batch mesophilic		16.2% increase	[252]

#### Combined methods

Combination of two more pretreatments methods have been experimented; for instance, combination of alkaline and enzymatic pretreatment has been evaluated with cassava peels for the production of bio-ethanol before biogas production; and it was reported that combined pretreatment improved biogas released by about 56% when equate to the control [273]. The application of combined pretreatments has been suggested by several researchers. Combination of different pretreatments was studied by Matsakas et al. [43]; organosolv alone, organosolv, and dilute acid, and combination of organosolv, dilute acid, and cellulolytic enzymes. It was reported by the authors that treatment duration was shortened and the highest yield was recorded when the three pretreatment was combined. Combined pretreatment of steam explosion, size reduction, and NaOH was experimented on *Miscanthus lutarioriparius* during biogas optimization. It was reported that combining steam explosion with 0.3 M NaOH and 0.5 mm particle size improved methane released by 57% when compared with the untreated substrate [274]. Sugarcane bagasse was pretreated with combine treatment of 1.0% acetic acid and steam explosion at 180 °C for 17.45 min reaction time. The optimum biogas yield was 434.47 L/kg VS which was about 91.88% improvement when compared with control experiment that produced 226.42 L/kg VS biogas yield [216]. In another related research, organic municipal solid waste was pretreated with microwave irradiation and NaOH before anaerobic digestion in semi-continuous mesophilic digesters. Microwave pretreatment at temperature of 35 °C with 60 mL of alkaline (20 meq NaOH/L) improved the solubilization of the feedstock by 53.2% compared to untreated feedstock. Biogas yield from pretreated substrate was increased by 205% in comparison to the control that was not treated [275]. It was noticed from this experiment that combined alkali and microwave irradiation pretreatment is effective in enhancing biogas yield from sewage sludge under mesophilic temperature. The application of combined alkaline and low-temperature pretreatment to improve biogas yield of waste activated sludge was investigated by Yi et al. [276]. It was reported that when 0.05 g NaOH/g TS was added to the substrate with a constant temperature of 70 °C for 9 h, 72.8% soluble carbohydrate/total carbohydrate was noticed, while biogas yield was six times higher than the control with average methane content of 64%. However, it has to be put into consideration that the higher the number of pretreatment methods used, the higher the expenses incurred in the process and this will make the process uneconomical and will not be able to contend with fossil fuels. Hence, it is important to unravel simple, non-expensive and effective pretreatments that are sustainable and economical. Table 7 shows some applications of combined pretreatment and their effects on biogas yield.

**Table 7:** Different combined pretreatment applied to the biogas production and yield

S/N	Biomass	Pretreatment	Anaerobic digestion condition	*Y*_BP_	*Y*_AP_	Refs
1	Park waste and cattle dung	Size reduction + alkali + fungal	Batch at 35 °C	102.6 L/kg VS	125.9 L/kg VS	[277]
2	Wheat straw	Ensiling + fungal	1 L Serum flask at 35 °C		66% increase in biogas yield	[278]
3	Rice straw	NaOH + hydrothermal	Batch at 37 °C	59.8 L/kg VS (CH_4_)	132.7 L/kg VS (CH_4_)	[109]
4	Cassava residue	Thermal + H_2_SO_4_		158 mL/g VS	248 mL/g VS	[279]
5	Sugar cane bagasse	Ethanol + NH_3_	1.18 L Bioreactor at 35 °C	105.6 mL/g VS	299.3 mL/g VS	[280]
6	Wheat Straw	NH_3_ (0.7%) + thermal (55 °C)	0.25 L reactor at 55 °C	407.8 mL/g VS	491.7 mL/g VS	[281]
7	Wheat Straw	NH_3_ (0.7%) + thermal (105 °C)	0.25 L reactor at 55 °C	407.8 mL/g VS	538.1 mL/g VS	[281]
8	Sludge biomass	Fenton + ultrasonic	250 mL Serum bottle at 35 °C	0.16 gCOD/g COD	0.17 gCOD/g COD	[282]
9	WAS	CaCl_2_ + bacteria	Batch reactor at 35 °C	0.145 L/g VS	0.322 L/g VS	[283]
10	WAS	Citric acid + ultrasonic	Batch reactor at 35 °C	0.212 L/g VS	0.433 L/g VS	[284]

## Discussion

Based on the literatures consulted, it can be noticed that there are various physical, chemical, biological, nano-particles, and thermal and combined pretreatments methods that has been established recently to overcome the challenges of biodigestion of lignocellulose feedstocks. It can be inferred that each of these pretreatment techniques have their merits and challenges, and it is only when feedstock composition and pretreatment technique are matched correctly that the aim of pretreatment can be achieved. Appropriate selection of these will enhanced the performance of the biodigestion and gas released. The required energy for some of these methods is an important factor, sometimes; those methods that needed low energy produce little improvement in terms of degradation and biogas yield when compared with the methods that required higher energy input, but this is not the case for all situation. Higher degradation of lignocellulose and improvement in recalcitrant of feedstocks releases higher biogas yield. Some of these pretreatment methods were noticed to improve degradation rate, but have little or no effect on biogas yield. Some of these techniques required very high investment cost, but do not have significant improvement of biogas yield that corresponds to the investment cost. There are several studies in this subject matter of recent, but there is still a huge vacuum to fill especially in bringing some of these techniques to a point where they are economically feasible. The production of inhibitory compounds and toxic materials is another big challenge noticed in some of the literatures consulted. The production of inhibitory compounds and toxic materials during pretreatment usually has negative impact on the biogas producing bacterial and biodigesters, respectively. This has been another challenge recorded that raise another concern, because some of the benefits recorded during pretreatment were eroded away during anaerobic digestion as a result of unfriendly nature of these materials to methane producing bacterial. It was observed that pretreating all substrates with one pretreatment technique is not naturalistic, because different feedstock was noticed to have response to different pretreatment method and this may not be economically viable or enhance the energy balance for feedstock with high degradation rates. Although, according to the available feedstocks and techniques, the suitable method can be employed, but an efficient and economical method that meets the expected needs of industry scale is less reported so far.

## Limitations to pretreatments

It has been reported that effectiveness of pretreatment methods relies on the composition of the feedstock. It is not easy to identify the pretreatment method(s) of lignocellulosic feedstock that will produce the optimum yields. Irrespective of the method to be used, the universal primary pretreatment is the particle-size reduction of the lignocellulosic material. Although, generally, it has been reported that mechanical pretreatment significantly improves methane released, but one of the short coming of the method is the incapability to break down the lignin, a major hindrance to bioavailability of carbohydrates for biodigestion. It was reported that lignocellulosic feedstock must be degraded to 1–2 mm to remove hindrances during hydrolysis; nevertheless, size reduction is a costly process that exhaust nearly 33% of the cumulative energy required for the entire process [285]. Taking into account the huge energy needs for mechanical pretreatment and exorbitant cost of energy, sustenance of the method is not economical. Therefore, cutting down the energy required and improving the effectiveness of milling and grinding of feedstock will be of help to enhance the economics of the entire process.

Considering chemical pretreatment, high cost of reagents, and additional operations like neutralization and the need for digesters with high resistance to corrosion are the identified limitations [286]. In addition, the production of inhibitory compounds is an important factor to be cared for, because it can hinder or forcibly lower the transformation effectiveness of hydrolysis of lignocellulosic materials to methane. Therefore, improving the effectiveness and reducing the production of inhibitory products through the combination of chemical reagents with lower concentration and other pretreatments can assist in minimizing the cost. For alkali pretreatment, it is efficient in lignin solubility and little amount of alkali residue is available in the treated feedstock supports in neutralizing the pH subdue during the acidogenesis stage of biodigestion process. Hence, alkali pretreatment is more suitable with succeeding anaerobic digestion when equate to acid pretreatment [287]. For organic solvents, the retrieving of every constituents of lignocellulosic feedstock is possible and this is an enhancement in the principal expenses in a biorefinery concept, but large quantity of downstream wastes and appropriate equipment are the limitations [288]. In extreme conditions, ionic liquids are more excellent for a broad range of utilization when compared to organic solvent [289], and nevertheless, the cost of the ions and necessity to recycle it are limitations. Some nano-particles have been reported to improve biogas yields and one of the main obstacles associated with this technology which is the price of biocatalyst has been eliminated through the fabrication of nanobiocatalysts. The major challenge now is the need for photo-digestion digesters that has visible-light photoactive metal oxides to improve the quantity of hydrogen released and accordingly increase the methane yield [290]. In thermal pretreatment, chemical is not always required and this will not be taken into account. Nevertheless, the liberation of unsuitable compounds like furfural, hydroxymethylfurfural, and phenolic acids due to high temperatures can hinder the process. This method is also suitable in locations where waste heat from a nearby factory or power plant is available as a means to minimize the expenses incurred on energy when heating [291]. Biological pretreatments produce low inhibitory compounds and generally the inhibition influence in the succeeding stage of anaerobic fermentation is minimal in comparison to chemical and physicochemical pretreatments. Despite having a lot of advantages, biological pretreatments also have some drawbacks like longer treatment duration, particular conditions of growth, larger space, and carbohydrate loss [286]. Exorbitant cost of fungi/enzymes/bacteria is a challenge to enhancing the economic reality of this process in biogas production [56, 240]. Table 8 shows some of the benefits and limitations of some pretreatment methods.

**Table 8:** Summary of some benefits and limitations of various lignocellulosic biomass pretreatment methods

S/N	Pretreatment methods	Benefits	Limitations
1	Mechanical milling	(i) It reduces the particle size and increases the available surface area (ii) Cellulose crystallinity of the lignocellulose is reduced (iii) The final particle size of the feedstock can be controlled (iv) It makes materials handling easy	(i) The energy required for this method is high and most times energy consumption is more than the energy released by the lignocellulose feedstock
2	Extrusion	(i) The fiber is shortened and defibrillated (ii) It releases low inhibitory products (iii) The method operates at higher solid loadings (iv) The treatment time required is short	(i) The method required high energy (ii) The impact of the method is limited without chemical addition (iii) It is mostly efficient on herbaceous lignocellulose materials
3	Pulsed electrical field	(i) It is operated at ambient conditions (ii) Lignocellulose cells are interrupted (iii) The equipment for the process is simple	(i) There is need for more research in this area because there is little information about it
4	Pyrolysis	(i) It releases both liquid and gaseous products	(i) It requires very high temperature (ii) The system produce ash (iii) Formation of inhibitory compound is possible
5	Acid hydrolysis	(i) Hemicelluloses are hydrolyzed to xylose and other sugars (ii) It modifies the lignin arrangement (iii) The acid itself is not needed always needed for enzymatic hydrolysis (iv) It hydrolyzes lignocellulose materials into fermentable sugars	(i) The cost of setting up the process is high (ii) Equipment with high resistance to corrosion is needed because of the corrosive properties of the acid (iii) The system produces toxic substances (iv) The process release inhibitory compounds
6	Alkaline hydrolysis	(i) It dislodges lignin and hemicellulose (ii) It improves the available surface area of the feedstock (iii) The sugar degeneration is lesser when compared with acid pretreatment	(i) The residence time needed is long (ii) The alkaline used is not recoverable (iii) The salts liberated during the process are consolidated into the feedstock (iv) The process liberated some inhibitors
7	Ozonolysis	(i) It reduces lignin component drastically (ii) The process does not generate toxic substances	(i) The quantity of ozone needed is high (ii) The method is expensive
8	CO_2_ explosion	It improves the available surface area of the feedstock It is economical It does not produce inhibitory substances	(i) It has no effect on lignin and hemicelluloses
9	AFEX	(i) It improves the available surface area (ii) Certain percentage of lignin and hemicellulose are eliminated (iii) The process does not produce inhibitory compounds that can affect the downstream process (iv) It is very effective and selective for reaction with lignin	(i) It is not effective for lignocellulose with higher percentage of lignin (ii) The effectiveness on softwood is poor (iii) High cost of ammonia and the environment is a major challenge
10	Biological	(i) It degrades both hemicellulose and lignin (ii) It requires low energy	(i) Hydrolysis rate is very low
11	Steam explosion	(i) It degrades hemicellulose and softens the lignin (ii) It is economical (iii) It required low initial capital investment (iv) It requires low energy (v) It has little or no effects on the environment	(i) The process destructs certain percentage of xylan (ii) The debasement of lignin-carbohydrate matrix is not complete (iii) Certain compounds that hinder the microorganisms are released (iv) It is not effective for softwood treatment
12	Deep eutectic solvents	(i) It removes lignin and some portions of hemicellulose (ii) It is a green solvent that is biodegradable and compatible	(i) The process is not effective when the pretreatment temperature is high
13	Supercritical fluids	(i) It removes the lignin and reduces the cellulose content (ii) The process does not degrade sugars since the solvent used is green (iii) This technique is appropriate for mobile biomass pretreatment processors	(i) The total costs of setting up the process are too high
14	Microbes	(i) It degrades lignin and hemicellulose selectively (ii) It is eco-friendly	(i) Due to slow yield, the process can take a longer period (several weeks)
15	Organosolv	(i) It dislodges lignin and hemicellulose disintegration (ii) It generate feedstock with low lignin residue that lower unneeded adsorption of enzymes (iii) The chemical can be retrieved and reuse	(i) The capital investment for the method is very high (ii) Difficulties in the handling of harsh organic solvents (iii) Inhibitory compounds are generated from this method
16	Oxidation	(i) It dislodges lignin and hemicelluloses (ii) It degrades cellulose partially (iii) By-products produced are usually low	(i) The cost of setting up the process is high
17	Ionic liquid	(i) It reduces the crystallinity and slightly removes hemicellulose and lignin (ii) The process requires low pressure solvent equipment (iii) The process takes place under modest reaction conditions	(i) High cost of the process (ii) Synthesis and purification of the liquid is complex (iii) The process is toxic (iv) Biodegradability is poor (v) An inhibitory compound that hinders enzymatic hydrolysis is released
18	Liquid hot water	(i) It eliminates soluble lignin and hemicelluloses	(i) The leftover lignin hinders the subsequent hydrolysis by the enzymes (ii) The quantity of water is needed (ii) It is energy intensive
19	SPORL	(i) It eliminates lignin (ii) It is efficient for softwood and hardwood (iii) It requires low energy	(i) It required size reduction prior to pretreatment and this size reduction required high energy
20	Hydrothermal	(i) It does not require the use of chemical (ii) It hydrate cellulose (iii) It removes hemicellulose part Certain percentage of lignin is removed	(i) There is formation of inhibition in the process (ii) High temperature is required
21	Combined process	(i) They are more effective than a single process	(i) They are often complex (ii) They are not always economical
22	Microaerobic	(i) It is considerably faster (ii) There is no process challenges associated with fibers and big chunks in the biogas digester	(i) Some of the organic matters that are supposed to be digested to methane are digested to carbon dioxide if the pretreatment time is too long (ii) The leach bed reactor needs to be emptied and the solid fractions removed of
23	Enzymes	(i) Higher substrate solubilization (ii) It is sustainable and ecological	(i) Relatively high price of enzymes for a limited improvement in biogas yield (ii) The process is slow
24	Ultrasound and irradiations	(i) It increases the pore size and surface area (ii) It disrupts feedstock crystallinity and reduces polymerization	(i) It consumes high electricity (ii) High cost of equipment (iii) Maintenance of the equipment is expensive
25	Nanoparticles	(i) It has high surface area to volume ratio (ii) It has high selectivity, specificity and potential catalytic activity (iii) It is an eco-friendly method (iv) It does not produce inhibitory compound	(i) Some nano-particles inhibit biogas producing microorganisms (ii) Operation cost is high (iii) Some nanoparticles are toxic (iv) They have low stability and reusability
26	Wet oxidation	(i) It is very effective in pretreatment of feedstock with high lignin content	(i) Cellulose is less affected (ii) Possibility of producing inhibitory products at high temperature is high (iii) It is not advisable at the industrial scale because of the combustible nature of oxygen and high cost of hydrogen peroxide used in the process
27	High hydrostatic pressure	(i) The pressure is distributed proportionally in all parts of the feedstock irrespective of its shape and size (ii) Pressure favors all structural reaction and changes that involve a decrease in volume	(i) Exorbitant cost of the equipment
28	High-pressure homogenizer	(i) It has high disintegration potential (ii) It requires minimal operation cost (iii) It is easy to operate and handle (iv) It does not require chemical addition	(i) The initial cost is high (ii) The method depends on the shear stress as a result of pressure gradient

## Conclusions

Increase in biofuels utilization will contribute to sustainable growth by cutting down the greenhouse gas released, sustainable management of wastes, and the utilization of nonrenewable resources. The use of lignocellulosic residues from agricultural and forest rather than traditional feedstock (starchy crops) could turn out to be a fantastic cost efficient and sufficiently accessible source of sugar for the production of fuel for transportation and other energy utilization. Nevertheless, the structural arrangements of lignocellulosic residues still exhibit technological hindrances as result of their restriction to bio-accessibility, and pretreatment of this refractory feedstock is crucial to enhance the accomplishment of anaerobic digestion process. Available surface area, crystallinity of cellulose, lignin guard, and casing by hemicellulose all lead to the hindrance of cellulose in feedstock during hydrolysis. The feedstock pretreatment and the inner arrangement of the feedstock itself are majorly accountable for their succeeding hydrolysis. The considerations used in the selection of pretreatment method will have effect on different feedstock characteristic, which will determine the availability of the feedstock to hydrolysis and the succeeding digestion of the sugars released. Hence, feedstock pretreatment is a very crucial step in the production of biogas from lignocellulosic materials, and is very important to comprehend the basics of the different processes that can assist in deciding the appropriate method with regards to the structural arrangement of the feedstock and the hydrolysis executor. Nevertheless, as earlier stated, the main limitations are the energy cost, cost of operation, and production of inhibitory compounds that affects the downstream bioprocess of producing biogas and other value-added products significantly. Although, it seems that combining different pretreatments presents a feasible solution, it is required to be specific on the type of feedstock and the downstream bioprocess type to generate bioenergy and other ancillary products. To accomplish a techno-economic possibility of using lignocellulosic materials, the idea of incorporated biorefiniries where two or more bio-products are produced in the same platform can be a bright concept. The incorporation of hydrogen, bio-ethanol, or biodiesel refinery into biogas refinery will lower the cost of lignocellulose pretreatment and add one or more valuable stream into the biorefinery. This will also encourage lignocellulose pretreatment in industrial scale significantly. Waste from this added refinery can be the feedstock for biogas production thereby, eliminate pretreatment of the feedstock. The two-stage process of hydrogen production before methane production will be more productive compared to one stage of methane production alone. The use of wastes from amino acids and lipids extraction process is suggested to improve the biogas yields from lignocellulose materials, since the waste would have undergone pretreatment before anaerobic digestion. One of the major challenges of ethanol production from biomass is the utilization of the left over wastes from the process. The use of these wastes in the incorporated biorefineries will eliminate both the challenges of pretreatment of lignocellulose during biogas production and the challenges of waste management in ethanol production. Some of the methods considered in the literatures were investigated at the laboratory scale experiments and this may not have the same yield when tried at the commercial scale. Therefore, there is need to investigate these methods at the commercial scale and come up with the best methods of lignocellulose pretreatment for commercial purposes. This review can assist as an influential instrument for the subsequent research on pretreatment of feedstock for biogas yields enhancement.

## Data Availability

Not applicable.
